# Effects of *Amphidinium carterae* Phytocompounds on Proliferation and the Epithelial–Mesenchymal Transition Process in T98G Glioblastoma Cells

**DOI:** 10.3390/md23040173

**Published:** 2025-04-16

**Authors:** Julia Oyón Díaz de Cerio, Giulia Venneri, Ida Orefice, Martina Forestiero, Carlos Roman Baena, Gianluca Bruno Tassone, Isabella Percopo, Angela Sardo, Maria Luisa Panno, Francesca Giordano, Valeria Di Dato

**Affiliations:** 1 Ecosustainable Marine Biotechnology Department, Stazione Zoologica Anton Dohrn Napoli, 80133 Naples, Italy; juliaoyon12@gmail.com (J.O.D.d.C.); ida.orefice@szn.it (I.O.); carlos.roman.baena@imbrsea.eu (C.R.B.); angela.sardo@szn.it (A.S.); 2 Department of Pharmacy, Health and Nutritional Sciences, University of Calabria, 87036 Rende, Italy; giuliavenneri.311@gmail.com (G.V.); martina.forestiero@unical.it (M.F.); gianluca.tassone@yahoo.it (G.B.T.); mluisa.panno@unical.it (M.L.P.); 3 Department of Research Infrastructures for Marine Biological Resources, Stazione Zoologica Anton Dohrn Napoli, 80122 Naples, Italy; isabella.percopo@szn.it

**Keywords:** glioblastoma, T98G, EMT, dinoflagellate, *Amphidinium carterae*, secondary metabolites, E-cadherin, vimentin, N-cadherin, α-SMA, twist

## Abstract

Glioblastoma (GBM) is an aggressive type of brain cancer, frequently invasive, with a low survival rate and complicated treatment. Recent studies have shown the modulation of epithelial–mesenchymal transition (EMT) biomarkers in glioblastoma cells associated with tumor progression, chemoresistance, and relapse after treatment. GBM handlings are based on aggressive chemical therapies and surgical resection with poor percentage of survival, boosting the search for more specific remedies. Marine eukaryotic microalgae are rapidly advancing as a source of anticancer drugs due to their ability to produce potent secondary metabolites with biological activity. Among such microalgae, dinoflagellates, belonging to the species *Amphidinium carterae*, are known producers of neurotoxins and cytotoxic compounds. We tested the capability of chemical extracts from two different strains of *A. carterae* to modulate the EMT markers in T98G, human GBM cells. In vitro proliferation and migration studies and EMT biomarkers’ abundance and modulation assays showed that the different *A. carterae* strains differently modulated both EMT markers and the proliferation/migration capability of GBM cells. This study sets the bases to find a marine microalgae-derived natural compound that could potentially target the epithelial–mesenchymal transition in brain-derived tumor types.

## 1. Introduction

Glioblastoma (GBM) is the most common primary malignant brain tumor. Despite having a relatively low incidence, if untreated, the median survival time is 3 months, and the survival percentage, in diagnosed patients, is estimated to be less than 5% over 5 years post-diagnosis [1,2,3]. This drastic low rate of survival is intrinsic to the GBM invasiveness capacity and localization in brain nuclei involving the control of speech, motor functions, and senses, complicating its extensive surgical resection. As a consequence, the post-surgery treatment include radiotherapy and concurrent strong repetition of chemotherapy oral cycles [2].

GBM cells, present in the neuroepithelial environment, exhibit a mesenchymal phenotype. However, markers involved in the regulation of the key processes of invasion and metastasis can induce the transformation of GBM cells into an invasive mesenchymal (MES) subtype [4,5,6]. The key process involved in the regulation of invasion and metastasis is the epithelial–mesenchymal transition (EMT) that transforms the epithelial cells into a mesenchymal type through the loss of cell polarity and cell–cell adhesion properties while acquiring migratory capabilities by expressing cell-surface and cytoskeletal proteins [7,8]. Despite being responsible for the processes inducing the acquisition of the cancer stem cell phenotype, EMT is involved in physiological processes, such as embryonic development, wound healing processes, and tissue formation [7]. It allows the cells to detach from the primary site; survive in the body fluids; enter and exit the blood vessel; seat and reacquire the epithelial phenotype; and resist stresses (hypoxia, nutrient depletion, and mechanical constrains) and different kinds of therapies. This mechanism is regulated by miRNA (microRNA) and a specific combination of transcription factors (TFs) from the Snail (Snail Family Transcriptional Repressor 1), Zeb (Zinc Finger E-Box Binding Homeobox), and Twist (Twist Family BHLH Transcription Factor) family, in cooperation with the oncogenes RAS (Rat sarcoma virus), N-MYC (avian myelocytomatosis viral oncogene neuroblastoma-derived homolog), p53 (Tumor protein P53), and cyclin-dependent kinase inhibitors [8]. Commonly recognized EMT biomarkers are E-cadherin (Epithelial–cadherin, CDH1) and N-cadherin (Neuronal–cadherin, CDH2) from the cadherin protein family, together with Vimentin (Vim) from the intermediate filament (IF) protein family. E-cadherin, responsible for the epithelial phenotype, is repressed by those TFs binding on different sites of the E-cadherin promoter, thus inducing the loss of tight cell junctions and the transformation in mesenchymal type cells. On the other hand, the same TFs activate the expression of the mesenchymal phenotype markers N-cadherin and Vimentin [9,10]. The N-cadherin (CDH2) counterpart is a cell–cell adhesion molecule, critical in cell attachment, and is considered a marker of mesenchymal cells [11]. Most studies have indicated that a reduction in E-cadherin (CDH1) expression is a defining feature of EMT [12,13]. Indeed, proteolytic cleavage, chromosomal deletions, epigenetic regulation, and transcriptional silencing of the CDH1 promoter prevent E-cadherin function and lead to the development of several epithelial malignancies [12,13].

Although the contribution of EMT in glioma progression is not as clear as that in epithelial tumors, the glial–mesenchymal transition has been revealed be an essential process in glioma invasion, contributing to tumor progression, chemoresistance, and relapse after treatment [5,14]. For the most part, GBM mesenchymal subtypes, expressing neuronal stem cell markers, resemble the EMT process and are characterized by high aggressiveness. Also, in gliomas, EMT is characterized by the reversible activation of the same TFs named above through intricate regulation balancing their opposite roles in both contrasting and stimulating the EMT carcinogenic process [7,8]. Twist and Snail family members have shown the ability to increase GBM cell motility and invasiveness both in vitro and in vivo [15,16,17]. Indeed, in GBM-derived cell lines, Twist1 mediates the alteration of cell–cell adhesions and actin cytoskeleton structures [15], while Snail1 and Snail2 are responsible of the migratory and invasiveness properties of malignant gliomas [16,17]. Although the majority of them do not demonstrate intrinsic E-cadherin expression, this has been observed for a small subset of highly malignant E-cadherin-positive GBMs [18]. On the other hand, N-cadherin’s role in glioblastoma is still debated, but recent studies show its involvement in glioblastoma progression, with an increased expression in glioma stem cells resistant to radiotherapy [11]. Vimentin, the major cytoskeletal component of mesenchymal cells, has been associated with the malignant progression of tumors of the central nervous system [19,20,21], becoming a molecular marker for enhanced migration and invasion of glioma cells [22]. α-SMA (alpha smooth muscle actin) is an isoform of actin and is an epithelial to mesenchymal transition (EMT) markers [23]. Considering such contrasting evidence on GBM molecular mechanisms and unsuccessful treatments associated with a low rate of patient survival, the necessity to find appropriate effective pharmacological treatments is evident. In this context, marine microalgae can represent a good source of potential compounds that are active against GBM. Indeed, many marine microalgae-derived bioactive compounds have the potential to target a wide range of pathological processes [24,25,26], such as oxidation, inflammation, cancers, microbial infections, and diabetes [27,28,29,30]. Due to the challenging conditions offered by the sea with different grades of both biotic (nutrient availability) and abiotic factors (pressure, light, temperature, salinity, competitors, predators, and pathogens), marine microalgae produce different amounts of secondary metabolites that help them to thrive within multiple environments [31]. Among such marine microalgae, dinoflagellates are a consistent group of single-celled micro-eukaryotes, mostly known for their ability to synthesize neurotoxins and to cause harmful algal blooms (HABs) [32,33]. Similar to other classes of marine eukaryotic microalgae, they are able to synthesize a diverse variety of lipid-derived secondary metabolites, such as sterols, macrocyclides, polyketides, and prostaglandins with bioactive properties that are important for human wellbeing [34,35,36]. Thus they have been studied as a sustainable source of bioactivity, mostly focusing on their pharmacological applications [24]. Examples come from Okadaic acid, which represents one of the bioactive compounds produced by dinoflagellates, with cytotoxic effects against some cancer cells, such as leukemia [28] or lung adenocarcinoma [37], and Eribulin, a macrocyclic ketone attributed to dinoflagellates, which is a successful example of a marine-derived natural compound that has been approved for clinical trials [38]. With these premises, natural compounds from marine organisms could also have great hidden potential for acting against neuronal-derived tumor types that could support their therapeutic success. To verify the effectiveness of dinoflagellate-derived molecules against GBM, we decided to study the effect of different types of chemical extracts from two strains of the dinoflagellate species *Amphidinium carterae*.

*A. carterae* has shown anticancer potential by producing amphidinolides and other long-chain compounds with cytotoxic activity [28,39,40]. Here, we present a study of the effects of chemical extracts from two *A. carterae* strains on human GBM cells. The strains used, named FE102 (Francesco Esposito 102) and VL (Villa Lauro, Naples), differed for the site of isolation (the Atlantic Ocean and the Mediterranean Sea, respectively), offering diverse living conditions of temperature, light, nutrients, salt concentrations, and pollution. Cell viability, cycle, and migration were inhibited by the extracts of both strains with different intensities. EMT markers’ expression and protein levels were also modulated differently by the extracts of the two strains.

## 2. Results

### 2.1. Inhibition of Cell Proliferation

**Figure 1:**
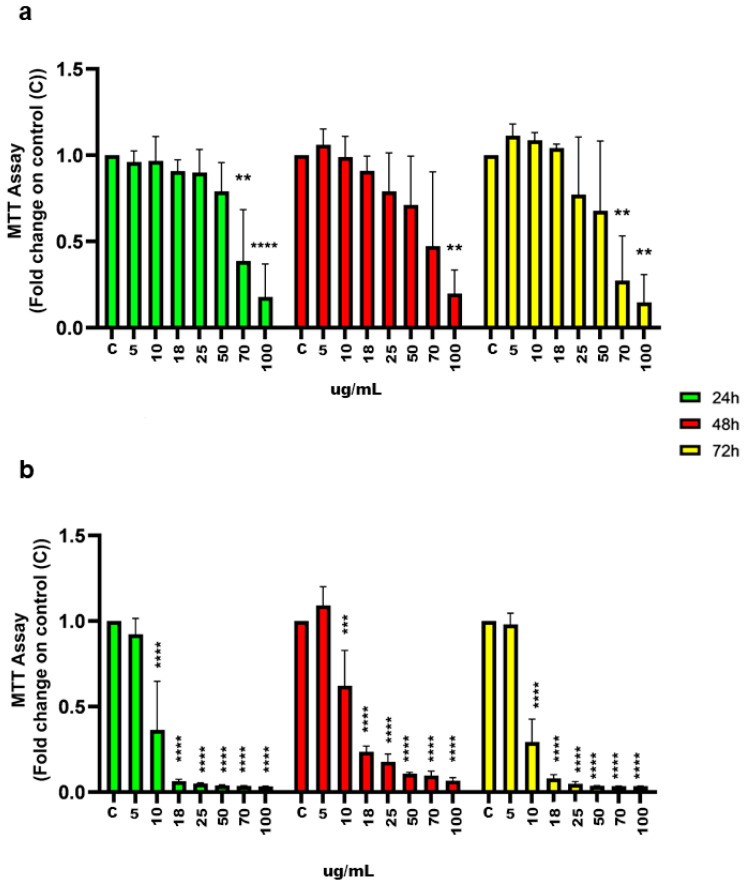
MTT (3-(4, 5-dimethylthiazolyl-2)-2, 5-diphenyltetrazolium bromide) assay: effects of *A. carterae* strains’ (VL and FE102) total extracts on T98G glioblastoma cell proliferation. (**a**) Strain VL; (**b**) strain FE102. Cells were treated with increasing doses of each extract for 24 h, 48 h, and 72 h. Cell proliferation is expressed as fold changes ± S.D., relative to C cells. Statistical analysis was performed by one-way Analysis of Variance (ANOVA), and the Bonferroni post hoc test was used to compare data (** *p* < 0.005; *** *p* < 0.0005; **** *p* < 0.0001).

Figure 1 shows a dose-dependent impairment of T98G (fibroblast-like cell from human glioblastoma multiforme) cell viability by *A. carterae* total extracts, with differences among the strains and time points tested. After 24 hours (h) of treatment, 70 µg/mL of the VL strain total extract significantly reduced T98G cells’ viability relative to the control (C) (Figure 1a). The inhibitory effect on cell viability, mentioned previously, was lost after 48 h, when a higher concentration was required, equal to 100 μg/mL, to obtain significant differences. However, longer treatments, 72 h, re-established the inhibitory effect at a 70 μg/mL concentration (Figure 1a). On the other hand, a significantly lower concentration of the FE102 strain total extract, 18 µg/mL, was sufficient to strongly inhibit T98G cells’ viability at all time points tested (Figure 1b).

**Figure 2:**
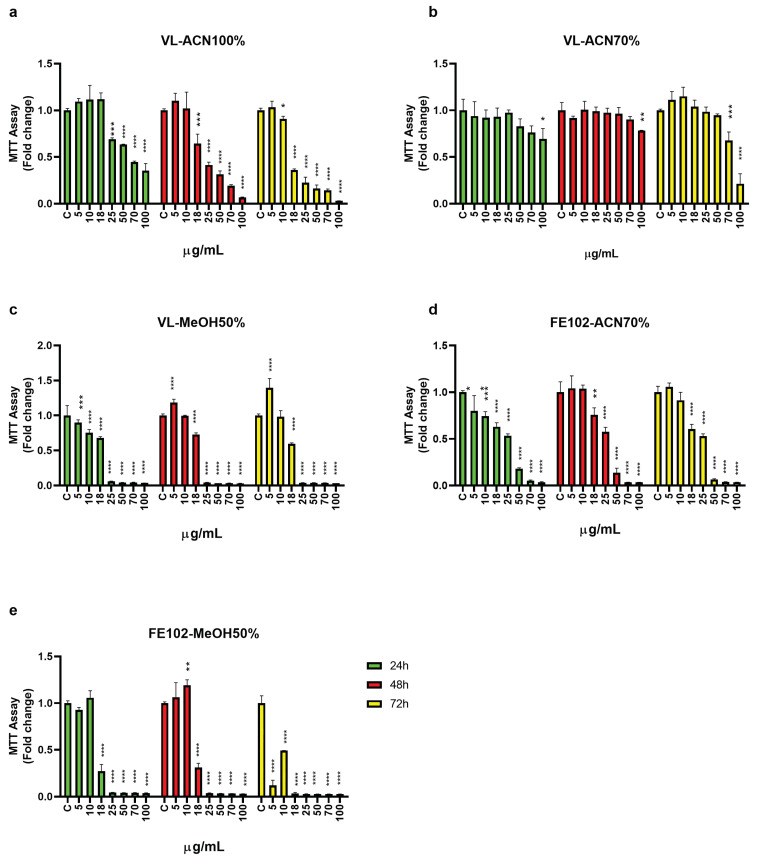
MTT assay: effects of fractionated extracts of *A. carterae* strains VL and FE102 on T98G glioblastoma cell proliferation. (**a**) VL-ACN 100%; (**b**) VL-ACN 70%; (**c**) VL-MeOH 50%; (**d**) FE102-ACN 70%; (**e**) FE102-MeOH 50%. Abbreviations: MeOH = methanol; ACN = acetonitrile. Cells were treated with increasing doses of the fractionated extracts for 24 h (green), 48 h (red), and 72 h (yellow). Cell proliferation is expressed as fold changes ± S.D., relative to C cells. Statistical analysis was performed by one-way ANOVA, and the Bonferroni post hoc test was used to compare data (* *p* < 0.05 ** *p* < 0.005; *** *p* < 0.0005; **** *p* < 0.0001).

Figure 2 shows the T98G cell viability when treated with different chemical fractionations of the two strains’ total extracts. VL fractionated with 100% acetonitrile (ACN), VL and FE102 fractionated with 70% ACN, and FE102 fractionated with 50% methanol (MeOH), induced a dose-dependent inhibition in T98G cell viability (Figure 2a–e). With respect to longer treatments (e.g., 48 h and 72 h, which inhibited T98G cell viability at 18 µg/mL and 10 µg/mL, respectively), the 24 h treatment with 25 µg/mL VL-ACN 100% caused a stronger inhibition of T98G cell viability (Figure 2a). VL fractionated with 70% ACN had an inhibitory effect on cell viability only with the highest concentration tested, 100 µg/mL, when cell treatment lasted 24 and 48 h. A lower concentration, 70 µg/mL, significantly impaired cell viability when treatment lasted longer, i.e., 72 h (Figure 2b). The same type of fraction (70% ACN) of the other strain, FE102, proved to be stronger, being able to significantly inhibit T98G cell viability at a concentration of 5 µg/mL when the treatment lasted 24 h, while to obtain the same effect with longer treatments, 48 h and 72 h, a slightly higher concentration equal to 18 µg/mL was necessary (Figure 2d). On the contrary, the VL-MeOH 50% fractions were stronger than the FE102 ones, being effective at a concentration of 5 µg/mL, independent of the duration of the treatment (Figure 2c), while the FE102-MeOH 50% fractions started to be effective at 18 µg/mL at 24 h and 48 h of treatment and at 5 µg/mL when the treatment lasted 72 h (Figure 2e).

### 2.2. Cell Cycle Impairment

The IC_50_ concentration calculated for each total extract turned out to be toxic, causing an immediate detachment and demise of the cells (Supplementary Figure S1). Thus, a concentration of 5 μg/mL of FE102 and VL total extracts were chosen for the following experiments.

**Figure 3:**
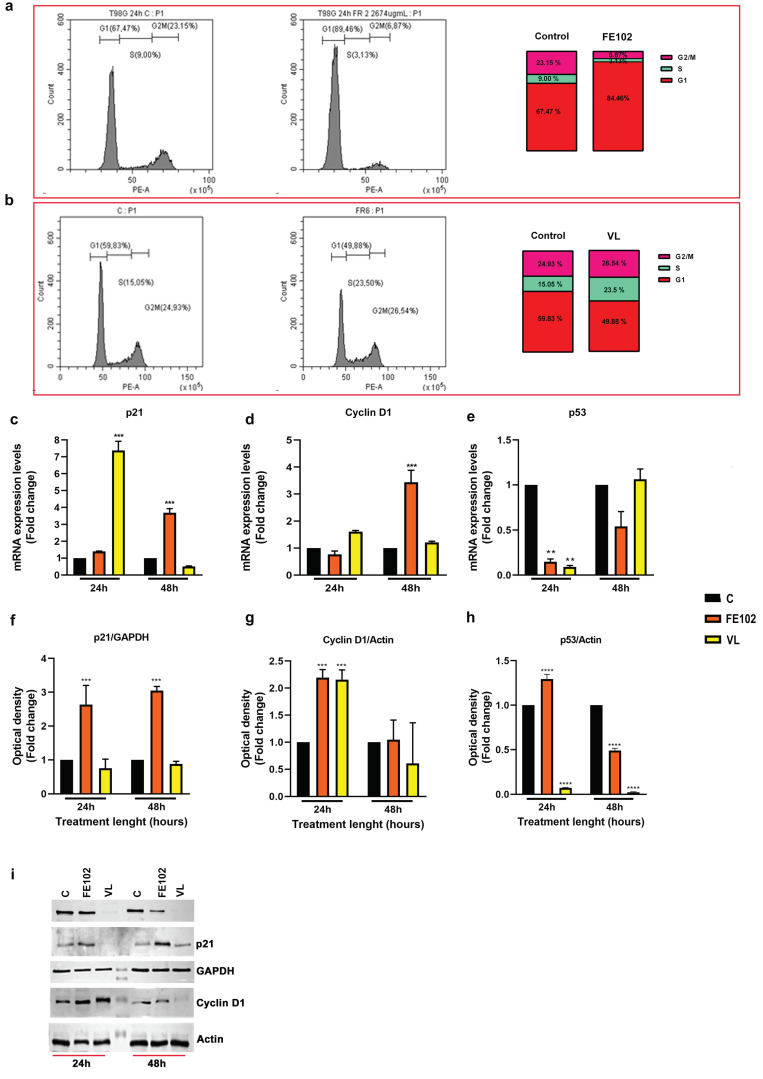
Effects of the *A. carterae* ACN 70% fractionated extracts on T98G cell cycle. (**a**) Cytofluorimetry analysis of the cell cycle phases following treatment with strain FE102 fractionated with 70% ACN. (**b**) Cytofluorimetry analysis of the cell cycle phases following treatment with strain VL fractionated with 70% ACN. (**c**) p21 gene expression levels expressed as folds on not treated cells (control) after 24 h and 48 h of treatment with FE102 or VL strains fractionated with 70% ACN; (**d**) cyclin D1 gene expression levels expressed as folds on not treated cells (control) after 24 h and 48 h of treatment with FE102 or VL strains fractionated with 70% ACN; (**e**) p53 gene expression levels expressed as folds on not treated cells (control) after 24 h and 48 h of treatment with FE102 or VL strains fractionated with 70% ACN; (**f**) optical densities of the corresponding p21 bands obtained by Western blotting after 24 h and 48 h of treatment with FE102 or VL strains fractionated with 70% ACN; (**g**) optical densities of the corresponding cyclin D1 bands obtained by Western blotting after 24 h and 48 h of treatment with FE102 or VL strains fractionated with 70% ACN; (**h**) optical densities of the corresponding p53 bands obtained by Western blotting after 24 h and 48 h of treatment with FE102 or VL strains fractionated with 70% ACN; (**i**) examples of protein bands obtained by WB related to p21, cyclin D1, p53, GAPDH (glyceraldehyde-3-phosphate dehydrogenase), and actin proteins. Bands were cropped from the original Western blotting (WB) membranes (see Supplementary Materials). Optical densities were calculated on protein bands derived from three independent WB hybridizations and are expressed as fold changes ± S.D., relative to control (not treated cells), after normalization with an internal reference protein (GAPDH or Actin). Statistical analysis was performed by two-way ANOVA, and the Bonferroni post hoc test was used to compare data (** *p* < 0.005; *** *p* < 0.0005; **** *p* < 0.0001). Significant differences, for each gene/protein, are reported only versus the control.

Treatment for 24 h with both total extracts did not impair the T98G cell cycle (Figure S2). However, the FE102 treatment resulted in a mild block of T98G cells in the G2/M (genomic/mitosis) phase (Figure S2b). On the contrary, VL and FE102 fractionated extracts showed the capacity to modulate the T98G cell cycle. Treatment for 24 h with the fraction FE102-ACN 70% resulted in a significant increase in cell portion in the G1 phase (Figure 3a). The expression levels of the cell cycle genes were regulated. p21 (cyclin-dependent kinase inhibitor 1A) and cyclin D1 (cyclin-dependent kinase 1) did not change, relative to C, after 24 h of treatment, while p53 (tumor suppressor p53) was significantly downregulated (Figure 3c–e). However, after 48 h of treatment, p21 and cyclin D1 significantly increased, by about 4-fold, relative to C, while p53 was raised, relative to C levels (Figure 3c–e). The corresponding protein levels of all three genes were already significantly upregulated, relative to C, after 24 h of treatment, but after 48 h, p21 remained significantly upregulated, cyclin D1 returned to C levels, and p53 was significantly downregulated.

On the other hand, the VL-ACN 70% treatment induced a slight increase in the S (synthesis) phase and no block in the G1 nor in the G2/M phase (Figure 3b). However, contrary to the treatment with the strain FE102, after 24 h of treatment, p21 was upregulated by 7-fold, relative to C, while cyclin D1 did not show any significant changes relative to C, and p53 was significantly downregulated. However, these were short-lasting regulations as after 48 h, all three genes returned to C levels (Figure 3c–e). p21 protein levels did not change relative to C neither after 24 h nor after 48 h of treatment (Figure 3f,i). The cyclin D1 protein was significantly upregulated after 24 h of treatment but returned to C levels after 48 h (Figure 3g,i). p53 remained significantly downregulated even after 48 h of treatment (Figure 3h,i).

**Figure 4:**
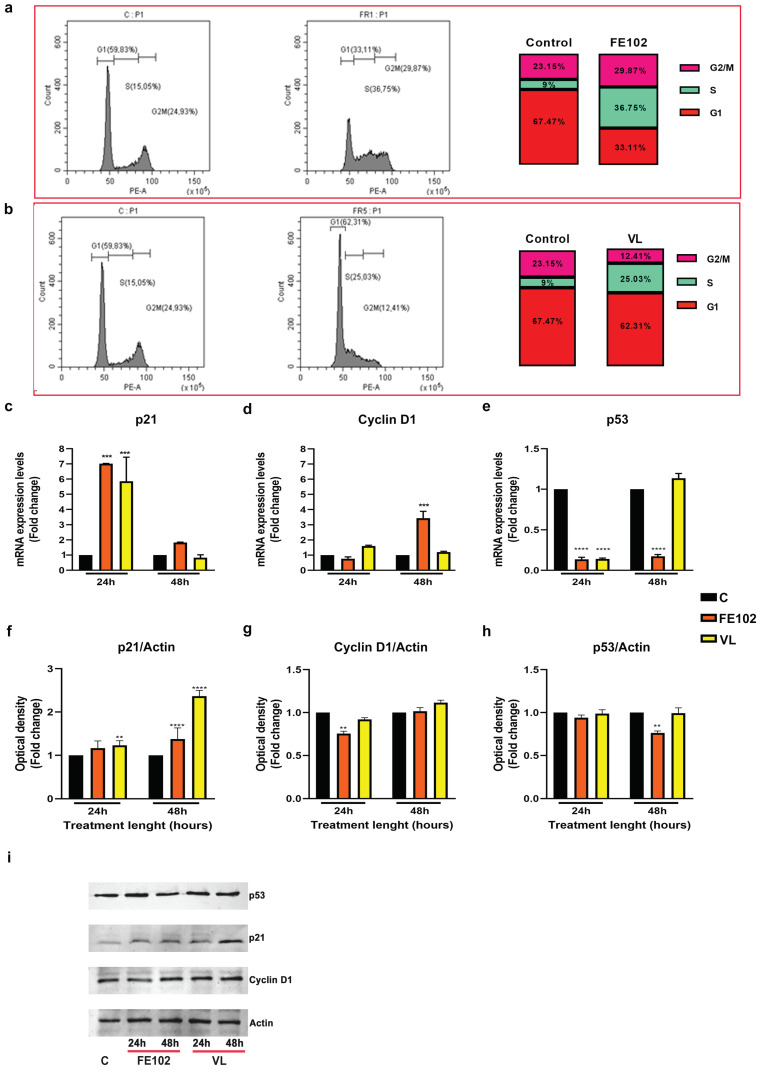
Effects of the *A. carterae* MeOH 50% fractionated extracts on T98G cell cycle. (**a**) Cytofluorimetry analysis of the cell cycle phases following treatment with strain FE102 fractionated with 50% MeOH. (**b**) Cytofluorimetry analysis of the cell cycle phases following treatment with strain VL fractionated with 50% MeOH; (**c**) p21 gene expression levels expressed as folds on not treated cells (control) after 24 h and 48 h of treatment with FE102 or VL strains fractionated with 50% MeOH; (**d**) cyclin D1 gene expression levels expressed as folds on not treated cells (control) after 24 h and 48 h of treatment with FE102 or VL strains fractionated with 50% MeOH; (**e**) p53 gene expression levels expressed as fold on not treated cells (control) after 24 h and 48 h of treatment with FE102 or VL strains fractionated with 50% MeOH; (**f**) optical densities of the corresponding p21 protein levels obtained by Western blotting after 24 h and 48 h of treatment with FE102 or VL strains fractionated with 50% MeOH; (**g**) optical densities of the corresponding cyclin D1 protein levels obtained by Western blotting after 24 h and 48 h of treatment with FE102 or VL strains fractionated with 50% MeOH; (**h**) optical densities of the corresponding p53 protein levels obtained by Western blotting after 24 h and 48 h of treatment with FE102 or VL strains fractionated with 50% MeOH; (**i**) examples of bands corresponding to protein levels obtained by WB related to p21, cyclin D1, p53, and actin proteins. Bands were cropped from the original Western blotting (WB) membranes (see Supplementary Materials). Optical densities were calculated on protein bands derived from three independent WB hybridizations and are expressed as fold changes ± S.D., relative to the control (not treated cells) after normalization on Actin, which was used as an internal reference protein. Statistical analysis was performed by two-way ANOVA, and the Bonferroni post hoc test was used to compare data (** *p* < 0.005; *** *p* < 0.0005; **** *p* < 0.0001). Significant differences, for each gene/protein, are reported only versus control.

Treatment for 24 h with both FE102- and VL-MeOH 50% induced an increase in the S phase, relative to C (Figure 4a,b), and an increase in p21 gene expression (Figure 4c) levels, which returned to those of C after 48 h of treatment. The protein levels slightly increased with both FE102 and VL fractions, especially during VL treatment (Figure 4f,i). Cyclin D1 gene expression levels increased significantly, by 3-fold (Figure 4d), relative to C, only after 48 h of treatment with the FE102-MeOH 50% fraction, but the protein levels remained stable, similar to those of C, throughout the treatments (24 h and 48 h) with both the FE102- and VL-MeOH 50% fractions (Figure 4f,g). At 24 h post treatment, p53 gene expression was significantly downregulated with both fractions, but at 48 h, under treatment with the VL strain, C levels were recovered (Figure 4e). The corresponding protein levels did not change, relative to C, except for at 48 h under treatment with the strain FE102, which resulted in a slight downregulation (Figure 4h,i).

### 2.3. Epythelial Mesenchimal Transition Modulation

To evaluate whether *A. carterae* strains were able to modulate the epithelial–mesenchymal transition (EMT) process, we evaluated the expression and corresponding protein accumulation of the genes involved in its mechanism: N-cadherin, Vimentin, α-SMA, Snail, and Twist.

**Figure 5:**
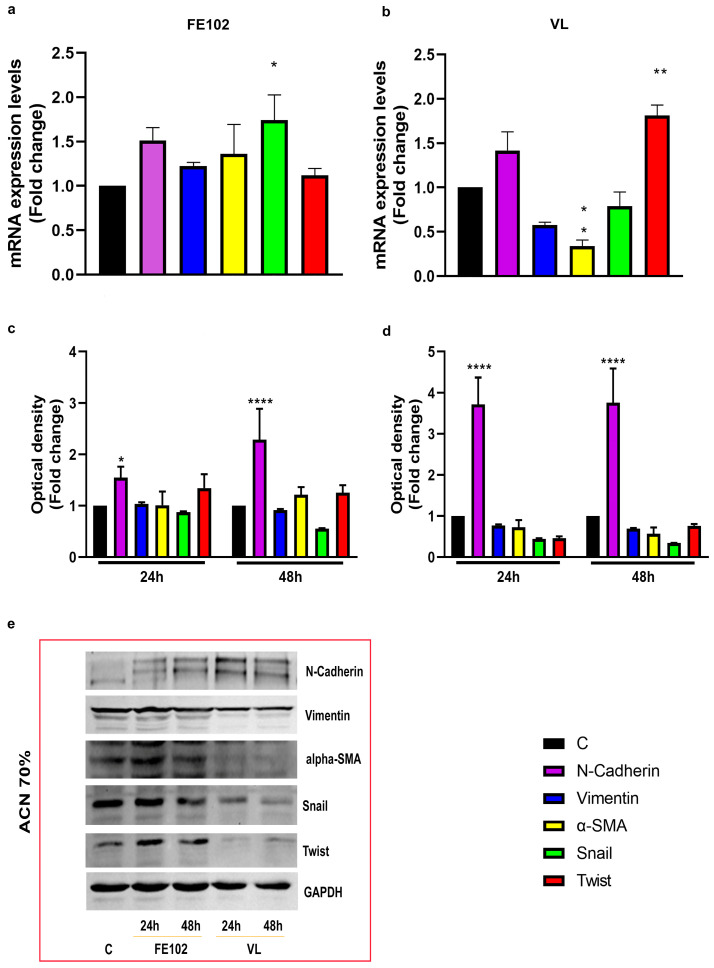
Effects of *A. carterae* ACN 70% fractionated extracts on EMT biomarkers. (**a**) Gene expression, represented as fold changes, relative to control, of the EMT’s genes after treatment with strain FE102-ACN 70% fractionated; (**b**) gene expression, represented as fold changes, relative to control, of EMT’s genes after treatment with VL-ACN 70%; (**c**) optical density of the EMT genes’ corresponding protein levels obtained by Western blotting after 24 h and 48 h of treatment with the strain FE102 fractionated with 70% ACN; (**d**) optical density of the EMT genes’ corresponding protein levels obtained by Western blotting after 24 h and 48 h of treatment with the strain VL fractionated with 70% ACN; (**e**) examples of bands corresponding to protein levels obtained by WB related to N-cadherin, Vimentin, α-SMA, Snail, Twist, and GAPDH (GlycerAldehyde-3-Phosphate Dehydrogenase) proteins. Bands were cropped from the original Western blotting (WB) membranes (see Supplementary Materials). Optical densities were calculated on the corresponding bands derived from three independent WB hybridizations and are expressed as fold changes ± S.D., relative to the control (not treated cells) after normalization on the GAPDH internal reference protein levels. Statistical analysis was performed by two-way ANOVA, and the Bonferroni post hoc test was used to compare data (* *p* <005; ** *p* < 0.005; ****p < 0.0001). Significant differences, for each gene/protein, are reported only versus control.

**Figure 6:**
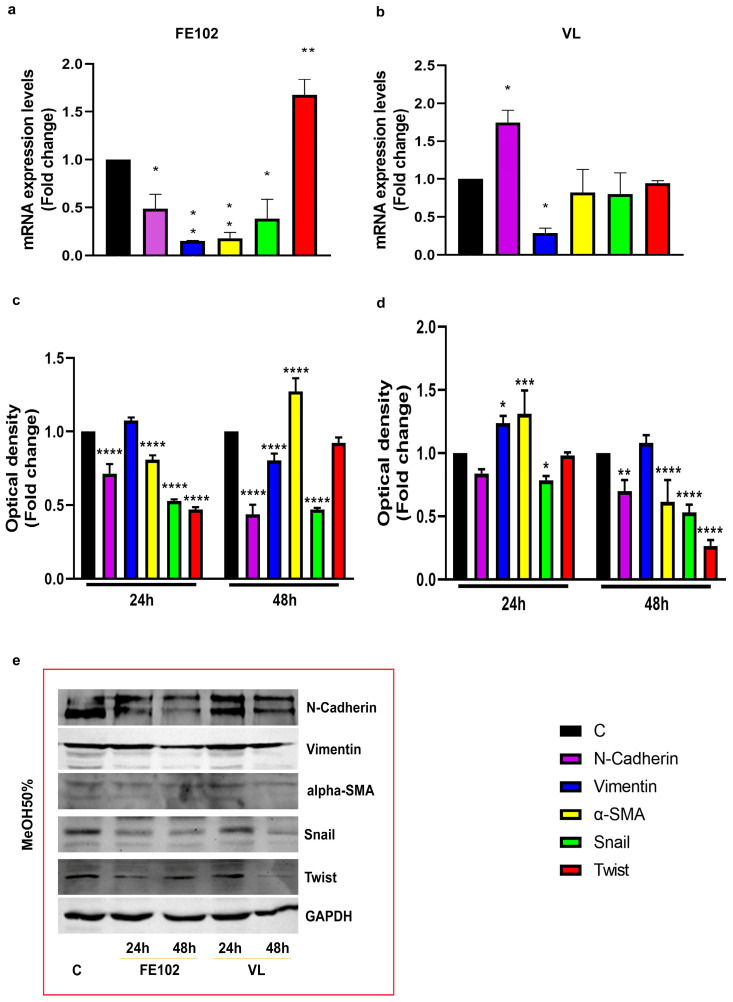
Effects of *A. carterae* MeOH 50% fractionated extracts on EMT biomarkers. (**a**) Gene expression, expressed represented as fold changes, relative to control, of the EMT’s genes after treatment with strain FE102 fractionated with 70% ACN; (**b**) gene expression, represented as fold changes relative to control, of EMT’s genes after treatment with VL-ACN 70%; (**c**) optical density of the corresponding EMT genes’ corresponding protein levels obtained by Western blotting after 24 h and 48 h of treatment with the strain FE102 fractionated with 70% ACN; (**d**) optical density of the corresponding EMT genes’ corresponding protein levels obtained by Western blotting after 24 h and 48 h of treatment with the strain VL fractionated with 70% ACN; (**e**) examples of bands corresponding to protein levels obtained by WB related to N-cadherin, Vimentin, α-SMA, Snail, Twist, and GAPDH (GlycerAldehyde-3-Phosphate Dehydrogenase) proteins. Bands were cropped from the original Western blotting (WB) membranes (see Supplementary Materials). Optical densities were calculated on the corresponding bands derived from three independent WB hybridizations and are expressed as fold changes ± S.D., relative to control (not treated cells), after normalization on the GAPDH internal reference protein levels. Statistical analysis was performed by two-way ANOVA, and the Bonferroni post hoc test was used to compare data (* *p* < 0.05; ** *p* < 0.005; *** *p* < 0.0005; **** *p* < 0.0001). Significant differences, for each gene/protein, are reported only versus control.

Different from the VL total extract, 24 h of treatment with the FE102 total extract significantly reduced the expression of all the markers tested, relative to C, particularly α-SMA (alpha-Smooth Muscle Actin) and N-cadherin (Supplementary Figure S2). Similarly, the fractionated extracts had different effects on both gene expression and protein levels of those markers (Figure 5 and Figure 6). FE102-ACN 70% induced a slight increase in Snail gene expression, but none of the marker’s protein levels were significantly impaired by this fraction (Figure 5a,c,e). The VL counterpart, downregulated α-SMA gene expression while upregulating the Twist one, but at protein level, only N-cadherin resulted in significant upregulation, relative to C, while the others appeared to not have changed (Vimentin) or to be inhibited (α-SMA, Snail, and Twist) (Figure 5b,d,e). FE102-MeOH 50% also significantly increased Snail gene expression while downregulating N-cadherin, Vimentin, α-SMA, and Snail gene expression (Figure 6a). The Vimentin and Snail protein levels were shown to be slightly downregulated, relative to C, after 24 h. Vimentin maintained lower levels also at 48 h, while Snail reached C values, and α-SMA decreased (Figure 6c,e). The VL-MeOH 50% fraction stimulated N-cadherin gene expression and inhibited the one of Vimentin while not having any effect on the gene expression of α-SMA, Snail, and Twist (Figure 6b). At protein levels, only Snail was shown to be downregulated, relative to C (Figure 6d,e).

### 2.4. Modulation of Cell Migration

**Figure 7:**
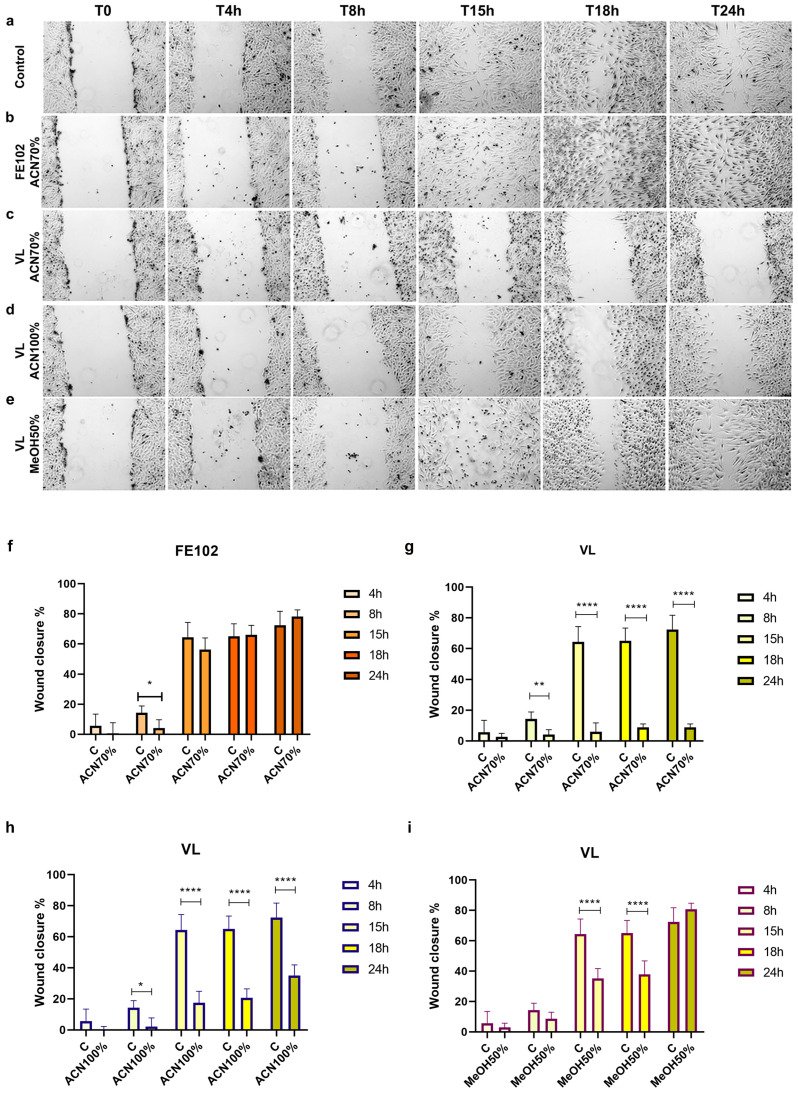
Cell migration inhibition by scratch-wound assay. (**a**–**e**) Photographs of wound closure evaluated at 4 h, 8 h, 15 h, 18 h, and 24 h post treatment. (**f**–**i**) Histograms showing the closure %, relative to the C, of cells treated with (**f**) FE102-ACN 70%, (**g**) VL-ACN 70%, (**h**) VL-ACN 100%, and (**i**) VL-MeOH 50%. The closure % was calculated on six different areas analyzed with the ImageJ softwarev1.54h. Statistical analysis was performed using one-way ANOVA, and the Bonferroni post hoc test was used to compare data (* *p* < 0.05; ** *p* < 0.01; **** *p* < 0.0001).

Both the FE102 and VL total extracts were able to inhibit T98G cell migration. However, FE102 exerted a more pronounced inhibitory effect, allowing for a 41.42% wound closure at 24 h of treatment relative to the 59.95% obtained with the strain VL (Supplementary Figure S3). The 70% ACN fraction of FE102 did not inhibit cell migration if not transiently at the beginning of the wound closure test, i.e., 4 h after the beginning of the test, relative to C (Figure 7a,b,f). The VL ACN counterparts, 70% (Figure 7a,c,g) and 100% (Figure 7a,d,h), significantly reduced cell migration at each time point tested (relative to C), while the corresponding −MeOH 50% fraction (Figure 7a,e,i) did so at the time points 15 h and 18 h (after the start of the wound closure test), after which the inhibitory effect was lost. The FE102-MeOH 50% fraction instead caused immediate cell detachment, preventing the execution of the test (personal communication).

## 3. Discussion

The epithelial–mesenchymal transition (EMT) is a flexible and dynamic mechanism that plays an essential role in various pathological processes, including wound healing, tissue fibrosis, and cancer progression [41]. It appears naturally in different tissue types and at different developmental stages [42] and is characterized by the creation of a spectrum of intermediate cellular states among the two main ones (called “partial-EMT” or “hybrid epithelial or mesenchymal phenotypes”), the epithelial and the mesenchymal phenotypes [43,44]. It confers multidrug resistance to tumor cells, remodels the tumor microenvironment into an immunosuppressed state, and is associated with a more aggressive tumor phenotype [41,45]. However, the EMT process can operate in both directions, epithelial to mesenchymal and mesenchymal to epithelial, often allowing cells to revert to epithelial phenotypes via the mesenchymal–epithelial transition (MET) [46].

GBM cells differ from typical epithelial cells due to the lack of a basement membrane within the neural environment and because of the inconsistent expression of E-cadherin, a typical marker of EMT [11,13]. Recent studies have shown that classical EMT markers’ regulation (upregulation of N-cadherin and Vimentin and downregulation of E-cadherin) [47] can prompt GBM cells to assume the invasive mesenchymal subtype (MES) [4], contributing to both tumor progression and chemoresistance, as well as recurrence following treatment.

Marine microalgae, such as dinoflagellates, populate all aquatic niches and are capable of synthesizing secondary metabolites, allowing them to adapt to different environments [48]. These secondary metabolites can have potential as high-value bioactive molecules for biotechnological applications [24,49], including pharmaceutical ones [50]. Some of them, indeed, were found to be effective against different tumor types [27,28,29,30]. The investigation of anticancer compounds derived from *A. carterae* covers a period of ca. 30 years, with the last anticancer compound, Amphidinol 22, characterized in 2022 from an ACN extract of the FE102 strain [39]. Cytotoxic activity against human skin melanoma and breast adenocarcinoma has been shown. Moreover, a strong cytotoxic activity of *A. carterae* extracts have also been shown for human leukemia cells (HL-60) [28,39,51].

In this work, we show the capability of this species to reduce cell viability and block the cell cycle advancement of glioblastoma T98G tumor cell lines. Our results show a different effect of the two strains’ extracts on T98G cells, confirming the clonal differences in secondary metabolites’ synthesis and concentration, which was already observed in other species, both under control conditions and under different abiotic parameters [35,52,53,54]. Notably, the extracts VL-ACN 100% and the FE102-ACN 70% demonstrated the most significant activity by gradually impairing cell viability in a dose- and time-dependent manner (MTT assay; Figure 2). Conversely, the other extracts did not impair the cell viability, gradually becoming 100% toxic a few hours after incubation with the extracts. However, the combined analysis of cell proliferation and cell cycle showed that the extract FE102-ACN 70% was the most effective in blocking the cell cycle in the G1 phase, supported by a long-lasting increase in p21 protein levels, thus preventing DNA duplication and cell duplication itself. On the other hand, VL-ACN 70% slightly blocked the cells in the S phase of the cell cycle with a 4-fold increase in cyclin D1 gene expression levels after 48 h of treatment, supported by an increase in the protein levels at 24 h but not at 48 h, while the p21 protein amount was not regulated, thus revealing no real impact on the cell cycle. Although the VL methanolic extract did not block the cell cycle, if not slightly in the S phase, it upregulated the p21 protein levels at 48 h while not regulating the cyclin D1 at both gene and protein levels. Thus, these data suggest that this extract also has the capability to block the cell cycle, which may involve other factors that have not been investigated in this work and that need more in-depth studies. On the other hand, the FE102 counterpart blocked the cell in the S phase and did not have any effect on p21 protein levels nor on cyclin D1, even if the corresponding gene expression levels were upregulated. Thus, the results with the VL extracts are in line with the presence of an anticarcinogenic compound acting through the p21 protein. Indeed, p21 competes with the Proliferating Cell Nuclear Antigen (PCNA) for binding with the DNA polymerase-δ and other proteins involved in DNA synthesis, thus directly inhibiting DNA synthesis [55]. These results are in accordance with the effect provoked by a significant group of anticancer drugs acting as cell mitosis inhibitors. By preventing the cell from dividing and thus entering the M phase of the cell cycle, the cancer cells stop replicating, and, as consequence, the tumor itself stops spreading [56,57]. Moreover, the p21 regulation was p53-independent (also due to a mutated p53 in the T98G cells), in accordance with previous reports showing the regulation of p21 expression by p53-independent mechanisms [58,59,60,61].

The aggressiveness of a tumor is not only determined by its capability to proliferate and grow but also by its capacity to penetrate the basement membrane and subsequently disseminate over long distances. The EMT process is regarded as the initial step in this process that culminates in metastatic dissemination, and in several studies, its activation has been proposed as the critical mechanism for the acquisition of a malignant phenotype [62,63,64,65,66,67]. It is characterized by a decrease in the expression of markers of the epithelial phenotype, including E-cadherin, the loss of intercellular junctions, and reduced intercellular adhesiveness. E-cadherin is a transmembrane protein involved in cell–cell adhesion, which is essential for maintaining tissue structure. In glioblastoma, its expression is often reduced or lost, which promotes a more invasive and migratory behavior in tumor cells [68,69]. This loss facilitates the epithelial-to-mesenchymal transition (EMT), enhancing the tumor’s ability to invade surrounding tissues and resist treatment. Consequently, decreased E-cadherin expression in glioblastoma is linked to poor prognosis and increased tumor aggressiveness [11]. This is frequently accompanied by increased cell motility and the expression of mesenchymal markers, such as Vimentin, an intermediate filament protein, and N-cadherin. Furthermore, the expression of other cytoplasmic proteins, including α-SMA, gamma (γ)-Actin, beta (β)-Filamin, and Thalin, as well as components of the extracellular matrix, such as fibronectin and collagen, have also been observed to be overexpressed [11,13,41,42,62,63,64,65,66,67]. Our treatments induced an overexpression of E-cadherin, relative to the control, after 24 h of treatment with both types of fractionations and strains, except for the strain FE102 fractionated with 70% ACN, whose effect lasted until 48 h (Figure S7). In addition, the EMT markers N-cadherin, α-SMA, Snail, Twist, and Vimentin were differently modulated. We observed contrasting effects on the up- or down-regulation of these markers, suggesting a blocking capability of the EMT process by our extracts but not an EMT reversal effect. The VL-MeOH 50% extract significantly reduced (*p* < 0.05) Vimentin expression but had no effect on α-SMA, Snail, and Twist, which closely approximated the gene expression levels observed in the untreated cells. However, the Snail protein levels were downregulated, suggesting an anti-EMT effect, considering that Snail has been associated with the promotion of drug resistance, tumor recurrence, and metastasis [70]. On the contrary, it augmented N-cadherin expression but not the corresponding protein levels, suggesting the capacity of this extract to stimulate a response against growth. Indeed, while this type of cadherin is widely regarded as a hallmark of the mesenchymal epithelium transition and as an indicator of ongoing EMT and various carcinoma developments, it is essential for the proper development of neuronal cells, and experiments on its functional loss seem to indicate a growth suppressor function [13].

On the other hand, the FE102-MeOH 50% extract downregulated the gene expression of four out of the five EMT markers, N-cadherin, α-SMA, Snail, and Vimentin, but, in contrast, upregulated Twist. Despite the gene expression activation, the corresponding protein levels were like those of C or were slightly decreased throughout the treatment period. The VL-MeOH 50% fraction induced the gene expression of N-cadherin and repressed that of Vimentin while not impacting that of α-SMA, Snail, and Twist. However, the respective protein levels remained like those of C or were slightly lower, except for the significant reduction in the Snail protein after 48 h of treatment. It is likely that these results suggest the presence, in our phytoextracts, of one or more compounds, with different polarities and bioactivities, which have the potential to slow the EMT process but not to cause a block. These different effects could be explained by the heterogeneity of the EMT state. Similar to our results, fucoxanthin [71] and phycocyanin [72], extracted from other microalgae, were able to arrest the EMT process, causing a less aggressive tumor phenotype. Moreover, spirulina-derived phycocyanin was able to down-regulate N-cadherin and Vimentin expression levels, similar to what we observed with the FE102 extracts.

NF-kB gene expression and protein levels and MMP-2 gene expression were downregulated or not changed under treatments with our extracts (Figures S5 and S6). Considering the known involvement of NF-kB in tumor development and the survival of migratory tumor cells in glioblastoma [73,74], these results further suggest the potential of the tested extracts to block tumor growth and metastasis dissemination.

Coherently, T98G migratory activity, a fundamental feature of the invasiveness of cancer cells, was also differently impaired by the tested extracts. In 2023, Wanyu Wang and colleagues also tested a natural compound, quercetin, for its capability of inhibiting T98G cell migration and found its effectiveness in the GBM treatment due to its capability of inducing apoptosis [75]. The fractions VL-ACN 70%, VL-ACN 100%, and VL-MeOH 50% markedly reduced the motility of T98G cells, thus indicating the potential of these compounds to mitigate the aggressive nature of glioblastoma. However, the VL-MeOH 50% extract effect did not last 24 h, while the ACN extracts significantly prevented the wound closure throughout the 24 h tested (*p* < 0.001). The short-lasting effect of the VL MeOH 50% extract could be due to a short half-life of its component compounds thus requiring repetitive treatments at 12 h intervals.

## 4. Materials and Methods

### 4.1. Cells and Strains

The *Amphidinium carterae* strain FE102 (Hulburt, 1957) was purchased from the NCMA collection (https://ncma.bigelow.org/CCMP121 accessed on 1 September 2023). The *A. carterae* strain Villa Lauro (VL) was isolated in the Gulf of Naples, near the coast, in a site known as Villa Lauro (Naples, Campania, Italy) in June 2019. Both strains were maintained in the microalgae collection of the Ecosustainable Marine Biotechnology Department of Stazione Zoologica Anton Dohrn Napoli and are freely available under request when needed.

T98G (T98-G, ATCC CRL-1690) is a “multiform” human glioblastoma cell line that was obtained from the American Type Culture Collection (ATCC) in March 2023. The experiments were conducted using cells that had undergone between six and ten passages.

### 4.2. Cell Culture and Harvesting

#### 4.2.1. *A. carterae*

Healthy, exponentially growing cultures (initial cell density: 5000 c/mL) of both strains of *A. carterae* were cultured in triplicates in polycarbonate carboys (S-B8160-2EA, Nalgene) containing 2 or 10 L of a K medium, prepared as described by Keller and co-workers [76]. The medium was prepared by using filtered (0.22 µm) sterile natural seawater, previously collected at −20 m of depth from oligotrophic zones of the Gulf of Naples (Mediterranean Sea, Italy), amended with appropriate amounts of the nutrient indicated in the culture medium recipe. Artificial light intensity (100 μmol m^−2^ s^−1^) was provided by daylight fluorescent tubes with a 12:12 h light:dark photoperiod. Dinoflagellate cultures were gently bubbled with sterile air (air flow: 2.5 L/min), provided by fill venting devices connected to 0.22 µm membrane pre-filters. Cell concentration was monitored daily through counts of Lugol-fixed samples in a Bürker chamber (depth 0.100 mm; Merck, Leuven, Belgium) under an inverted microscope (Axiovert 200, Zeiss, Oberkochen, Germany). At the onset of the stationary phase, the whole volume was harvested in a swing-out centrifuge (Allegra X-12R, Beckman Coulter GmbH, Krefeld, Germany) at 2300× *g* (10′, 4°C) to remove the supernatant. Algal wet biomasses were stored at -80°C until analyses.

#### 4.2.2. T98G

T98G cells were cultured in Corning^®^ tissue-culture treated culture dishes (35 mm × 10 mm, Merk—CLS430165-500EA) filled with 10 mL of the Minimum Essential Medium (MEM) 1× ((GIBCO) supplemented with 10% Fetal Bovine Serum (FBS) (Gibco—A5256801), 1% penicillin/streptomycin (Gibco—15140-122), 1% L-Glutamine (Gibco—25030-024), 1% Sodium Pyruvate (Gibco—11360070), and 1% non-essential amino acids (Gibco—11140050)) and grown in the dark at 37 °C and 5% CO_2_ in a CO_2_-supplemented incubator (Hera cell 150—Thermo, Milton Park, Abingdon, UK). Confluent plates were washed with 10× PBS (Gibco—70011044); detached with Trypsin (Gibco™ Trypsin 2.5%; no phenol red); and re-plated in new petri dishes at a dilution of 1:3.

Before each assay, growing cells were harvested by PBS washing and trypsin detachment and were counted using a Bürker–Türk chamber under a light microscope. The required number of cells, for each assay, was centrifuged at 1600× *g* for 5 min at 25 °C, resuspended in a suitable volume, and plated in 96-well multi-wells for MTT assays (BRAND^®^ microplate BRANDplates^®^, cellGrade—Merk—BR781962-50EA); in 6-well multi-wells for wound assays (Corning^®^ Transwell^®^ 6 well plates—Merk—CLS3450-24EA); and in 60 mm × 15 mm petri plates (Merk—CLS430166-500EA) for the protein and gene expressions.

### 4.3. Chemical Extraction

#### 4.3.1. Total Extract

A total of 2 L of the *A. carterae* cultures FE102 and VL were harvested on day 16 and 14, respectively, by centrifuging at 2300× *g* at 16 °C for 15 min (Eppendorf, Milan, Italy, 5810R). The 10 L cultures were collected as described above on days 14 and 13 for FE102 and VL, respectively. Each pellet was lyophilized at −80 °C and 0.1 atm using the Freeze Dryer Alpha 2-4 LSC plus (Christ, Germany).

Freeze-dried pellets were first resuspended in 10 mL per g of lyophilized biomass of 2:1 methanol (MeOH) (300-H410L, ROMIL GR.GRAD. SPS CAS (67-56-1))/chloroform (CHCl_3_) (300-H135L, ROMIL SPS × HPLC,UV,IR,PEST.CAS (67-66-3)); vortexed for 30 s; and then ice-sonicated (Bandelin, SONOPULS, Berlin, Germany) for 2 min at 20% power (30″ on—30″ off). Sonicated pellets were left for 30 min under mechanical stirring in the dark at room temperature and then centrifuged at 4 °C at 2990× *g* for 15 min (Eppendorf, Milan, Italy5810R). The supernatants were collected, while the remaining biomass was dissolved again in 10 mL per g of lyophilized biomass of the 2:1 MeOH/CHCl_3_ mix and again left under agitation in the dark for 30 min. The supernatant was again collected by centrifugation at 4 °C at 2990× *g* for 15 min (Eppendorf, 5810R, Milan, Italy). Both supernatants were then combined and dried using a rotavapor under an inert atmosphere using a nitrogen flush (Buchi, R-100, Flawil, Switzerland). Dried extracts were stored at −20 °C until further analysis.

#### 4.3.2. Total Extract Fractionation

Total chemical extracts derived from 10 L cultures of *A. carterae* were fractionated as described by Nuzzo et al. [41] with some modifications, as follows. The total chemical extracts were resuspended in 2 mL of MilliQ water and sonicated at 20% power for 5 min on ice. The sonicated solution was passed through a CHROMABOND empty column (3 mL, polypropylene, with PE filter elements; Macherey-Nagel); loaded with 2 g per 100 g of the total extract of Poly (styrene-divinylbenzene) (2% cross-linked, 200–400 mesh; Thermo Scientific Chemicals); and pre-activated with 20 mL of methanol 100% per mg of total extract followed by the same volume of MilliQ water. Once the sample was loaded in the column, 25 mL of MilliQ water per mg of total extract was used to wash the samples before the elution of each of the following fractions: MeOH 50% (1); acetonitrile (ACN) (300-H048L, ROMIL HPLC FAR UV-200 SPS, CAS (75-05-8)) 70% (2); ACN 100% (3); and dichloromethane (DCM) (300-H202L, ROMIL SPS × HPLC,UV,IR ETC. CAS (75-09-2))/MeOH 9:1 (*v*:*v*) (DCM) (4) (2 V of solvent per g of resin). The 4 eluted fractions were collected in different vials, dried under nitrogen flux with a rotavapor, and stored at −20 °C.

### 4.4. In Vitro Tests on Glioblastoma Cell Lines

The half-maximal inhibitory concentration (IC_50_) of each fraction was determined after the MTT assay (Section 2.1) using GraphPad Prism 8. The values of IC_50_ were then used for the following assays: cell cycle analysis (Section 2.2), gene expression studies by real-time qPCR (Section 2.3), and the cell migration assays (Section 2.4).

#### 4.4.1. Reagents

*A. carterae* extracts were resuspended into appropriate volumes of Dimethyl sulfoxide (DMSO) to reach a concentration of 10,000 μg/mL for the FE102 and VL total extracts and 1000 μg/mL for the FE102 and VL fractionated extracts. Table 1 lists the stock solutions and their working concentrations. The stock solutions were diluted in MEM (Minimum Essential Medium—ThermoFisher) to an intermediate concentration from which the working solutions for the cell treatments were derived. All extracts were checked for complete solubilization. A total of 1% of the total volume of DMSO that was used to resuspend each extract was used as a control (C) treatment in all the in-vitro assays performed.

For both strains, the 90% dichloromethane (DCM) fractions, upon resuspension in DMSO, formed lumpy precipitates, which prevented their use for further investigations. In addition, the FE102 100% acetonitrile (ACN 100%) fraction was also excluded from the analysis because of insufficient quantities.

#### 4.4.2. MTT Assay

T98G cells were plated in 96-well plates at a confluence of 5000 cells/well and were grown in complete media at 37 °C and 5% CO_2_ in a HERAcell 150 (ThermoFisher Scientific) incubator. Cells were starved in an FBS-free medium for 24 h, after which they were treated with increasing concentrations of each extract, from 5 μg/mL to 100 μg/mL, previously mixed with a full-red medium for 24, 48, and 72 h. At the end of each time point (24, 48, and 72 h), cells were washed and supplemented with fresh media added with 100 μL of 3-(4,5-dimethylthiazolyl-2)-2, 5-diphenyltetrazolium bromide (MTT) and were incubated for 3 h at 37 °C, 5% CO_2_. Cells were then washed and incubated for another 30 min at 37 °C, 5% CO_2_, in fresh media supplemented with 100 μL of DMSO (Dimethyl-sulfoxide). The reduction of MTT to formazan (resulting in a colored solution) by viable cells was quantified by measuring the absorbance at 500–600 nm using a 96-well plate spectrophotometer (Muliskan SkyHigh, ThermoFisher Scientific). The results are represented graphically, with the concentration of the substance employed as a treatment depicted on the x-axis and the fold change on the y-axis. This format enables the visualization of the dose–response relationship, thereby illustrating how cell viability expression varies as a function of the concentrations tested.

#### 4.4.3. Cell Cycle Analysis

A total of 10,000 cells/well were plated in 12-well plates (10 cm, Corning, SIAL, Italy) until 70–80% of confluence. Cells were starved with an FBS-free and phenol red-free medium for 24 h and then treated with 5 μg/mL of each extract of each strain for 24 h in a full-red medium. At the end of the incubation, the cells were pelleted, washed with phosphate-buffered saline (PBS), and resuspended in 500 μL of a 0.1% Propidium Iodide solution (Sigma Aldrich, St. Louis, MO, USA) supplemented with 1% octylphenoxypolyethoxyethanol (IGEPAL) (Sigma Aldrich) and 20 U/mL RNase-A (Sigma Aldrich). After 1 h of incubation, the DNA content was measured using a CytoFLEX flow cytometer (Beckman, Beckman Coulter, Milan, Italy). Data analysis was performed using the CytExpert Beckman Coulter softwarev2.4 (Beckman Coulter, Milan, Italy). The assay could not be performed with higher concentrations of the extract, because it caused the detachment and demise of the cells (Supplementary Figure S1).

#### 4.4.4. Gene Expression Studies by Real Time-Quantitative Polymerase Chain Reaction (rt-qPCR)

A total of 50,000 cells/well were plated in 60 mm plates until they reached a confluence of 70–80%. Cells were starved using an FBS-free and phenol red-free medium for 24 h and then treated with different doses of each extract for 24 h and 48 h. Table 2 lists the treatment concentrations.

Total RNA was extracted using TRIzol (TRIzol RNA Isolation Reagents—Thermo Fisher Scientific) following the manufacturer’s protocol. The total RNA concentration and purity was determined by spectrophotometric measurements at A260 nm, A260/280 nm, and A260/230 nm. The High-Capacity cDNA Reverse Transcription Kit (Thermo Fisher Scientific—K2562) was used to retro-transcribe 2 μg of the total RNA into cDNA.

A total of 1 µL of 1:10 diluted cDNA was used as a qPCR template in a 10 µL reaction mix composed as follows: 1 µL of the SYBR™ Green PCR Master Mix (Thermo Fisher Scientific), 7.8 μL of H_2_O RNase and DNase free, 0.1 µL of the 100 µM Reverse Primer and 0.1 µl of the 100 µM Forward Primer. cDNA was amplified at 50 °C for 2 min and at 95 °C for 2 min for 40 cycles at 95 °C for 15 s, at 60 °C for 15 s, and at 72 °C for 1 min. Analyses were conducted using the QuantStudio 1 Real-Time PCR Systems (ThermoFisher Scientific). The primers used for gene amplification are listed in Table 3. The 18S gene was used as a housekeeping gene for normalization, as it has been demonstrated to be a reliable reference gene in previous studies [64,65].

Gene expression levels were calculated by applying the comparative ΔCt method and are expressed as a fold change (FC) relative to the control (C). FC was calculated using the following formula:

(1) $$Relative\;fold\;change\;expression=2^{-∆(∆Ct)}$$

(2) $$∆Ct={Ct}_{target\;gene}-{Ct}_{housekeeping\;gene}$$

(3) $$∆∆Ct={∆Ct}_{target\;gene\;treated\;samples}-{∆Ct}_{target\;gene\;not\;treated\;samples}$$

Gene expression was assessed at 24 h, as gene transcription represents an early response to experimental stimuli. Typically, alterations in mRNA levels occur rapidly, providing an early indication of regulatory changes. However, protein expression is a later and more cumulative process, as it requires mRNA translation and, in many cases, post-translational modifications. Accordingly, protein levels were quantified at both 24 h and 48 h to facilitate direct comparisons with transcriptional data and to observe the temporal evolution of the protein-level response.

#### 4.4.5. Immunoblotting Analysis

T98G cells untreated (control, C) or treated with the different chemical extracts were lysed with 500 μL of the Radioimmunoprecipitation assay buffer (RIPA) (50 mM of Tris (hydroxymethyl) aminomethane (THAM) hydrochloride (Tris–HCl), 150 mM of Sodium Chloride (NaCl), 1% octylphenoxypolyethoxyethanol (NP-40), 0.5% sodium deoxycholate, 2 mM of sodium fluoride, 2 mM of Ethylenediaminetetraacetic acid (EDTA), 0.1% Sodium dodecyl sulfate (SDS)) with protease inhibitors (1.7 mg/mL of aprotinin, 1 mg/mL of leupeptin, 200 mmol/L of phenylmethylsulfonyl fluoride, 200 mmol/L of sodium orthovanadate, and 100 mmol/L of sodium fluoride; Sigma-Aldrich, Merck). Equal amounts of proteins were resolved on a SDS/polyacrylamide gel, transferred to a nitrocellulose membrane, and probed with primary antibodies against cyclin D1, p21, N-cadherin, Vimentin, Snail, α-SMA, Twist, GAPDH, and actin (Santa Cruz Biotechnology, DBA, Milan, Italy). At the end of the incubation, the membranes were washed and incubated with an alkaline Horseradish peroxidase (HRP)-conjugated secondary Immunoglobulin G (IgG) antibody. The antigen–antibody complex was revealed by HRP/H_2_O_2_-catalyzed oxidation of luminol in alkaline conditions using an enhanced chemiluminescence system (ECL, Santa Cruz Biotechnology, Milan, Italy).

#### 4.4.6. Scratch Assay

A total of 30,000 cells/well were plated in 6-well plates until 70–80% of confluence. Cells were starved for 24 h with an FBS-free and phenol red-free medium. Cells were scratched, washed two times in PBS to remove detached cells, and treated in a complete medium with different doses of each extract for 24 h. The treatment concentrations are listed in Table 4.

The treatment following the physical injury applied to the cell monolayer resulted in a toxic effect; thus, for this assay, it was not possible to use all the extracts used in the other assays. Likely, the simultaneous application of physical injury with the treatment was too strong, resulting in cell detachment.

Images were captured with the ImagineFocus Alpha softwarev4.5 at 3 h, 6 h, 9 h, 15 h, 18 h, and 24 h after the start of treatment (time zero). An analysis of wound closure was performed using the ImageJ software v1.54h.

### 4.5. Statistics

Data were analyzed to test statistical significance using one-way ANOVAs and Bonferroni post hoc tests. One-way ANOVA was used for unpaired data to test the probability of significant differences between the different groups of samples. Statistical tests were performed using the GraphPad Prism software (GraphPad Software, La Jolla, CA, USA).

## 5. Conclusions

The present study reports the ability of chemical extracts of two strains of *A. carterae* to inhibit glioblastoma T98G cell viability and cell cycle progression and motility, aside from modulating the expression of the genes responsible for the EMT process. Our findings suggest that the treatments in T98G glioblastoma cells induced a shift towards a more epithelial phenotype, as evidenced by the downregulation of mesenchymal genes and the upregulation of E-cadherin expression. This modulation of gene expression may indicate the reversal of the epithelial-to-mesenchymal transition (EMT), potentially enhancing cell–cell adhesion and reducing the invasive properties of the glioblastoma cells.

Follow-up studies will be needed to further fractionate the four active fractions, with the aim to isolate the pure compound(s) exhibiting bioactivity against glioblastoma cells. The available *A. carterae* genome will help with the in silico identification of secondary metabolites which could be responsible for the inhibitory effects on glioblastoma cell viability and cell cycle progression and migration capabilities. Moreover, the clonal differences presuppose different concentrations of bioactive metabolites that can be regulated by applying different abiotic growth conditions. This opens the way to biotech experimentation by testing more strains and conditions that induce a higher production of the bioactive metabolite/s, once identified by further fractionations coupled with Liquid Chromatography with tandem mass spectrometry (LC/MSMS) analysis.

To summarize, recognizing the lack of treatment options for neuronal cancer, the results presented in this manuscript might be the first step toward the discovery of microalgae-derived molecules which could be used as anti-glioblastoma treatments, but the lack of information regarding this and other dinoflagellate species suggest the need to perform further investigations.

## Figures and Tables

**Table 1:** Solutions and their stock concentrations.

Species	Strain	Type of Extract	Weight	Stock Solution	Working Stock
*Amphidinium carterae*	FE102	Total extract Replicate 1	100 mg	45,454 μg/mL	10,000 μg/mL
		Total extract Replicate 2	120 mg	54,545 μg/mL	10,000 μg/mL
		Total extract Replicate 3	80 mg	47,059 μg/mL	10,000 μg/mL
		MeOH 50%	3.1 mg	31,000 μg/mL	1000 μg/mL
		ACN 70%	3.5 mg	70,000 μg/mL	1000 μg/mL
		ACN 100%	1.3 mg	-	-
		DCM:MeOH 9:1	53.3 mg	-	-
	VL	Total extract Replicate 1	50 mg	50,000 μg/mL	10,000 μg/mL
		Total extract Replicate 2	20 mg	33,334 μg/mL	10,000 μg/mL
		Total extract Replicate 3	200 mg	117,647 μg/mL	10,000 μg/mL
		MeOH 50%	6.6 mg	29,000 μg/mL	1000 μg/mL
		ACN 70%	12.4 mg	62,000 μg/mL	1000 μg/mL
		ACN 100%	2.9 mg	58,000 μg/mL	1000 μg/mL
		DCM:MeOH 9:1	19.3 mg	-	-

Abbreviations: MeOH = methanol; ACN = acetonitrile; DCM = dichloromethane.

**Table 2:** Treatment concentrations used for gene expression studies by rt-qPCR.

*A. carterae* Strain	Type of Extract	Concentration
FE102	Total extract	5 μg/mL
	MeOH 50%	10 μg/mL
	ACN 70%	25.74 μg/mL
VL	Total extract	5 μg/mL
	MeOH 50%	18 μg/mL
	ACN 70%	18 μg/mL
	ACN 100%	18 μg/mL

Abbreviations: MeOH = methanol; ACN = acetonitrile.

**Table 3:** Oligo sequences used for the listed gene amplification by qPCR.

Oligo Name	Forward	Reverse
18S	5′-CGGCGACGACCCATTCGAAC-3′	5′GAATCGAACCCTGATTCCCCGTC-3′
P21F	5′ GACACAGGTGTTGTGACACAT-3′	5′CTCATTCAGCCTGACTTGGGA-3′
CYCLIN D1	5′-GATGCCAACCTCCTCAACGAC-3′	5′-CTCCTCGCACTTCTGTTCCTC-3′
N-CADHERIN	5′-ACAGTGGCCACCTACAAAGG-3′	5′-CCGAGATGGGGTTGATAATG-3′
VIMENTIN	5′-GAGAACTTTGCCGTTGAAGC-3′	5′-GCTTCCTGTAGGTGGCAATC-3′
TWIST1	5′-TCCAAATTCAAAGAAACAGGCG-3′	5′-CAGAATGCAGAGGTGTGAGGA-3′
SNAIL	5′-CGAGTGGTTCTTCTGCGCTA-3′	5′-GGGCTGCTGGAAGGTAAACT-3′
α-SMA	5′-AGACATCAGGGGGTGATGGT-3′	5′-CATGGCTGGGACATTGAAAG-3′

**Table 4:** Extract concentrations used to perform the scratch assays.

*A. carterae* Strain	Extract	Concentration
FE102	Total extract	5 μg/mL
	ACN 70%	25.74 μg/mL
VL	Total extract	5 μg/mL
	MeOH 50%	18 μg/mL
	ACN 70%	18 μg/mL
	ACN 100%	18 μg/mL

Abbreviations: MeOH = methanol; ACN = acetonitrile.

## Data Availability

The original contributions presented in this study are included in the article/Supplementary Materials. Further inquiries can be directed to the corresponding authors.

## Supplementary Materials

The following supporting information can be downloaded at: https://www.mdpi.com/article/10.3390/md23040173/s1, Figure S1: Toxic effect of the *A. carterae* FE102 and VL total extracts on the T98G glioblastoma cells; Figure S2: Effects of the *A. carterae* total extracts on T98G cells cycle; Figure S3: EMT markers gene expression under treatment with *A. carterae* total extracts; Figure S4: Scratch-wound assay to evaluate *A. carterae* total extracts migration inhibitory effect; Figure S5: NF-kB and MMP-2 gene expression; Figure S6: NF-kB protein levels; Figure S7: E-Cadherin gene expression.

## Abbreviations

The following abbreviations are used in this manuscript:

T98G	Human glioblastoma multiforme fibroblast cells
*A. carterae*	*Amphidinium carterae*
FE102	FE (Francesco Esposito) 102
VL	A. carterae strain VL (Villa Lauro)
GBM	Glioblastoma
EMT	Epithelial–mesenchymal transition
MET	Mesenchymal–epithelial transition
HAB	Harmful algal bloom
CDH1, E-cadherin	Epithelial cadherin
CDH2, N-cadherin	Neuronal cadherin
α-SMA	Alpha smooth muscle actin
GAPDH	Glyceraldehyde-3-phosphate dehydrogenase
MTT	3-[4,5-dimethylthiazol-2-yl]-2,5 diphenyl tetrazolium bromide
PCNA	Proliferating cell nuclear antigen
DNA	DeoxyriboNucleic acid
IF	Intermediate filament
K medium	Keller medium
PBS	Phosphate buffered saline
CO_2_	Carbon dioxide
FBS	Fetal bovine serum
A	Absorbance
cDNA	Coding DNA
Atm	Atmosphere
h	Hour
V	Volume
g	Microgram
mL	Milliliters
L	Liter
Mm	Millimeter
Mg	Milligram
cm	Centimeter
U	Unit
min	Minute
°C	Centigrade
TP	Time point
FC	Fold change
ACN	Acetonittile
MeOH	Methanol
CHCl_3_	Chloroform
DCM	DiChloroMethane
IC_50_	Inhibitory concentration
C	Control
MEM	Minimum essential medium
DMSO	Dimethyl sulfoxide
Tris–HCl	Tris(hydroxymethyl)aminomethane hydrochloride
NaCl	Sodium chloride
EDTA	(Ethylenedinitrilo) tetraacetic acid
SDS	Sodium dodecyl sulfate
RIPA	Radio-immunoprecipitation assay
HRP	Horseradish peroxidase
ECL	Enhanced chemiluminescence
PCR	Polymerase chain reaction
qPCR	Quantitative polymerase chain reaction
ANOVA	Analysis of variance
J.O.D.	Julia Oyón Díaz de Cerio
G.V.	Giulia Venneri
I.O.	Ida Orefice
M.F.	Martina Forestiero
C.R.B.	Carlos Roman Baena
G.B.T.	Gianluca Bruno Tassone
I.P.	Isabella Percopo
A.S.	Angela Sardo
M.L.P.	Maria Luisa Panno
F.G.	Francesca Giordano
V.D.D.	Valeria Di Dato
