# Supplementary material for: Effects of Amphidinium carterae Phytocompounds on Proliferation and the Epithelial–Mesenchymal Transition Process in T98G Glioblastoma Cells
Source: Mar Drugs. 2025 Apr 16;23(4):173. doi: 10.3390/md23040173 (PMC12029094; doi:10.3390/md23040173)
Supplement: Supplementary file 1 [file marinedrugs-23-00173-s001.zip › marinedrugs-3526997-supplementary.pdf]

Supplementary Materials

Authors: Julia Oyón Diaz de Cerio, Giulia Venneri, Ida Orefice, Martina Forestiero, Carlos Roman Baena, Gianluca Tassone, Isabella Percopo, Angela Sardo, Maria Luisa Panno, Francesca Giordano and Valeria Di Dato.

Title Pages

Methods 2

IC50 determination...2

Table S1 2

Figure S1. Toxic effect of the A. carterae FE102 and VL total extracts on the T98G glioblastoma cells............3

Figure S2. Effects of the *A. carterae* total extracts on T98G cells cycle...4

Figure S3. EMT markers gene expression under treatment with *A. carterae* total extracts.............................4

Figure S4. Scratch-wound assay to evaluate *A. carterae* total extracts migration inhibitory effect 5

Original wound assay microscope Images... 6-11

Original Western Blotting Images 12-13

Figure S5. NF-kB and MMP-2 gene expression...14

Figure S6. NF-kB protein levels...15

Figure S7. E-Cadherin gene expression...16

Methods

IC_{50} determination

The cell viability was determined through the MTT assay using different extract's concentrations. The IC_{50} value relative to each extract tested was calculated using the GraphPad programme.

Table S1: IC_{50} concentrations.
Missing values correspond to extracts not suspendable in DMSO.

**Strain**	**Extract**	**IC50** (μg/mL)
VL	Total extract	-
	MeOH 50%	18 μg/mL
	ACN 70%	18 μg/mL
	ACN 100%	18,14 μg/mL
	90% DCM 10% MeOH	-
FE102	Total extract	-
	MeOH 50%	10 μg/mL
	ACN 70%	25,74 μg/mL
	ACN 100%	-
	90% DCM 10% MeOH	-

A. carterae total extract turned out to be toxic (Figure S1), thus for the subsequent experiments a lower working concentration equal to 5g/ml, not provoking a toxic effect, was chosen.

a.

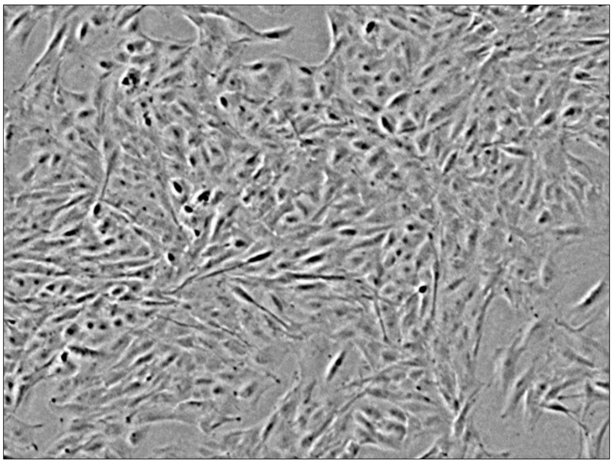

b.

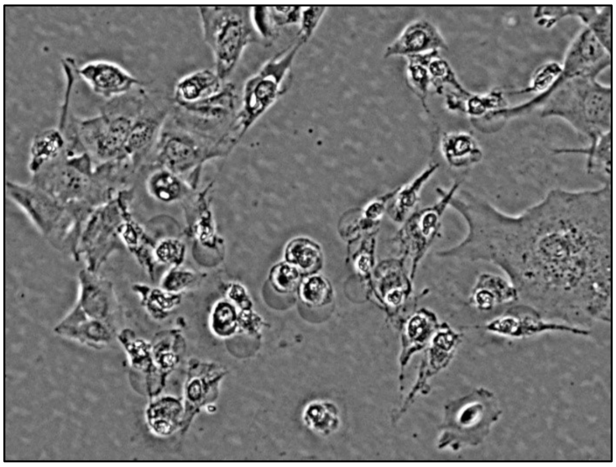

c.

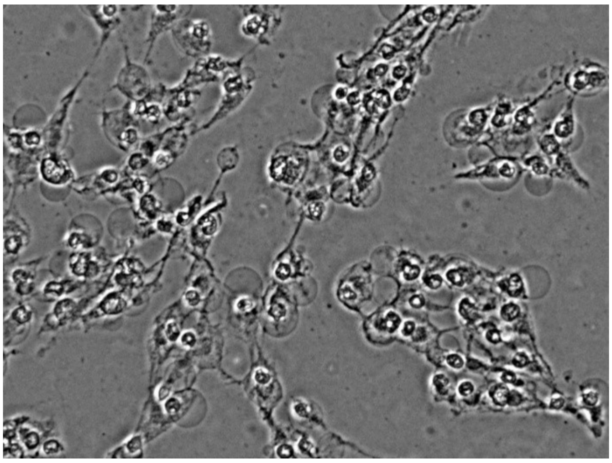

Figure S1. Toxic effect of the *A. carterae* FE102 and VL total extracts on the T98G glioblastoma cells. Detachment and demise of the cells is clearly visible in respect to untreated cells. **a.** Untreated cells (Control); **b.** T98G cells treated with *A. c.*-VL total extract; **c.** T98G cells treated with *A. c.*-FE102 total extract. Pictures imaging: Optical microscope, 10x magnification**.**

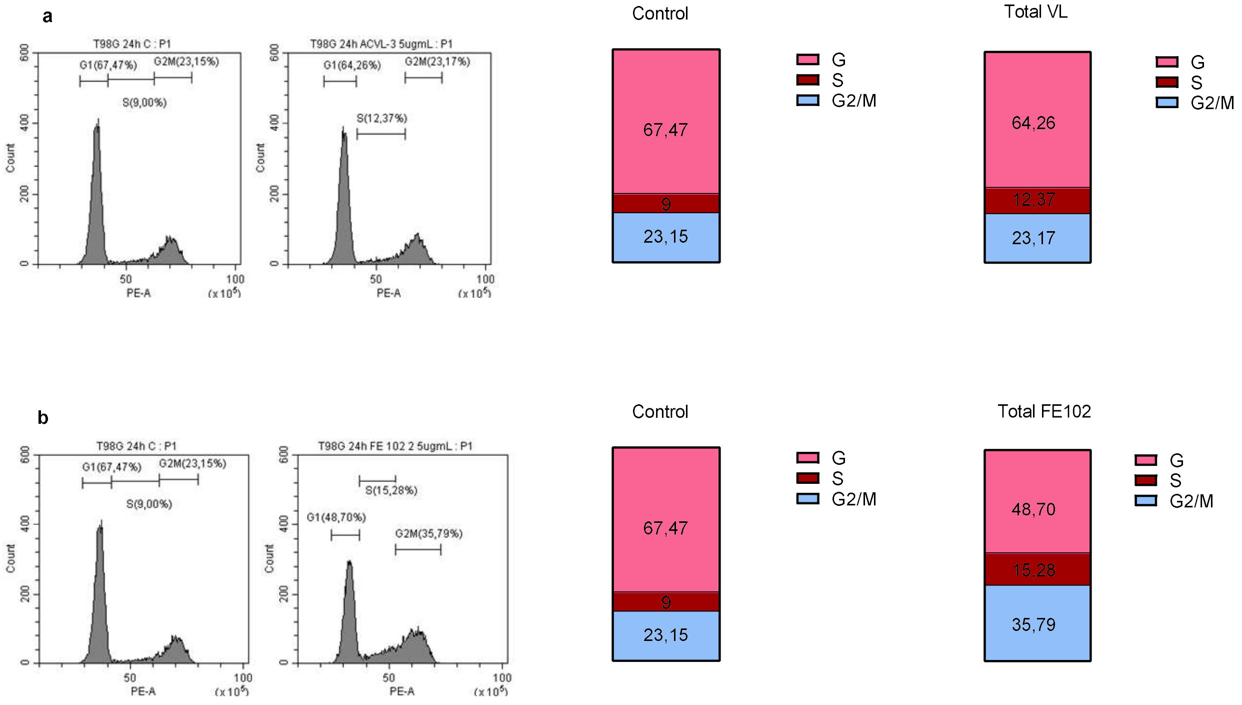

Figure S2. Effects of the *A. carterae* total extracts on T98G cells cycle.
a. Analysis of cell cycle phases by cytofluorimetry following treatment with VL total extract; **b.** Analysis of cell cycle phases by cytofluorimetry following treatment with FE102 total extract.

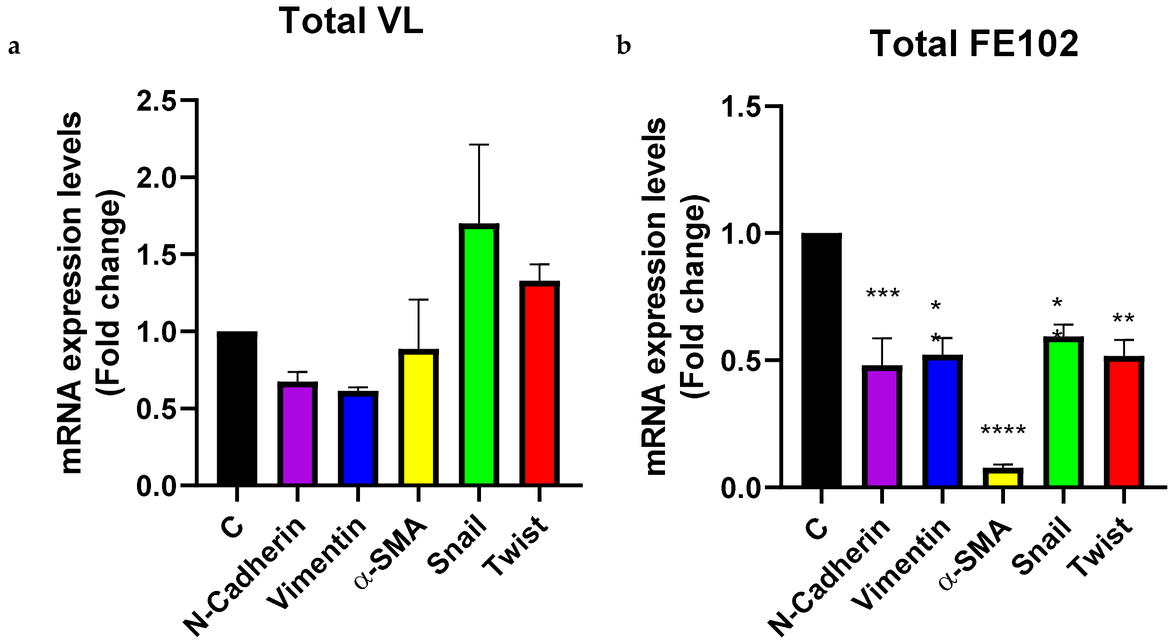

Figure S3. EMT markers gene expression under treatment with *A. carterae* total extracts. a. mRNA expression of EMT's genes after treatment with VL total extract; **b.** mRNA expression of EMT's genes after treatment with FE102 total extract. Statistical analysis was performed by two-way ANOVA, and the Bonferroni post hoc test was used to compare data (*p<0.05 ****p<0,0001).

a

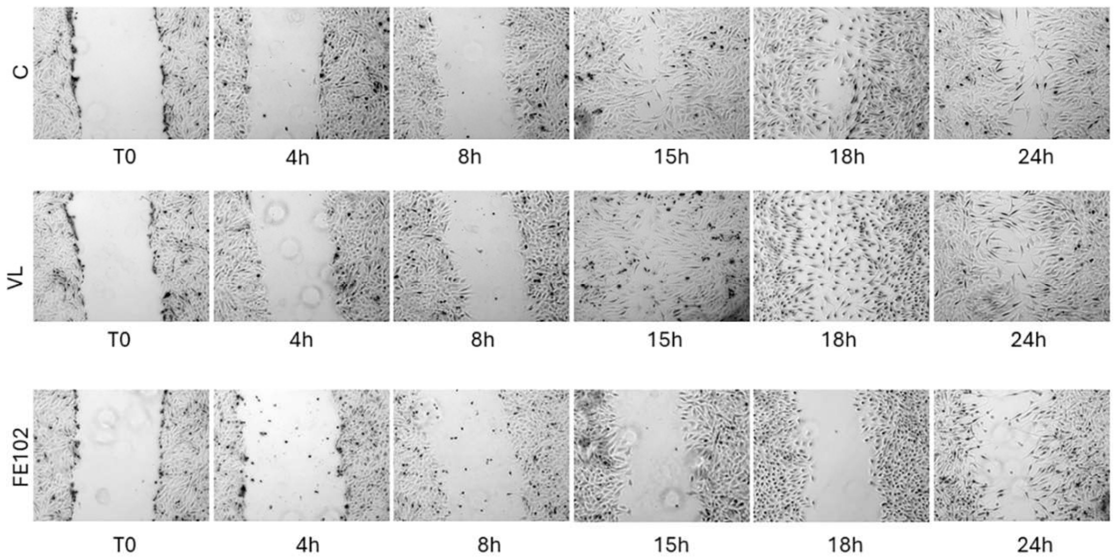

b

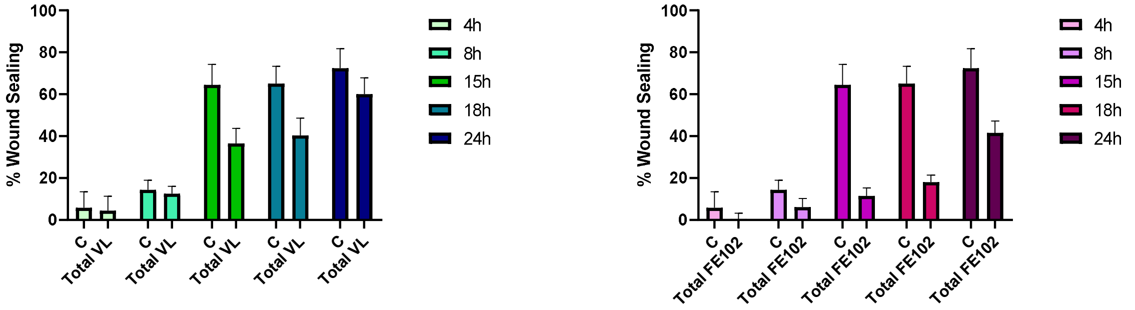

Figure S4. Scratch-wound assay to evaluate *A. carterae* total extracts migration inhibitory effect.
a. Images taken at 4h, 8h, 15h, 18h and 24h post injury applied with different treatments; **b.** Percentage of wound closure at each time point after VL total extract treatment; **c.** Percentage of wound closure at each time point after FE102 total extract treatment. The closure % of treated cells vs Control cells was calculated with ImageJ using six different areas. Statistical analysis was performed using One-way Anova- Bonferroni.

Original wound assay microscope images

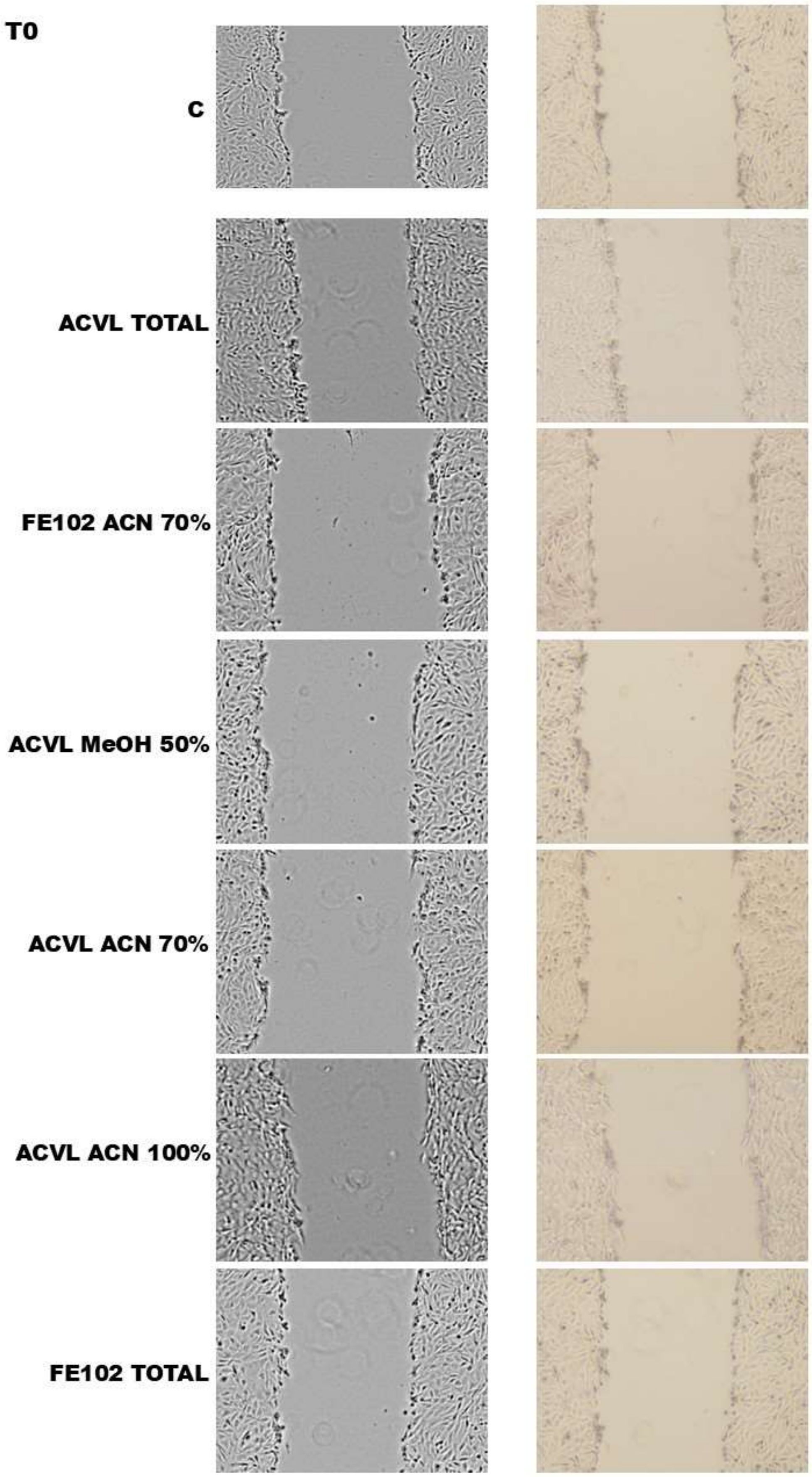

4H

C

ACVL TOTAL

ACVL ACN 70%

ACVL ACN 100%

ACVL MeOH 50%

FE102 TOTAL

FE102 ACN 70%

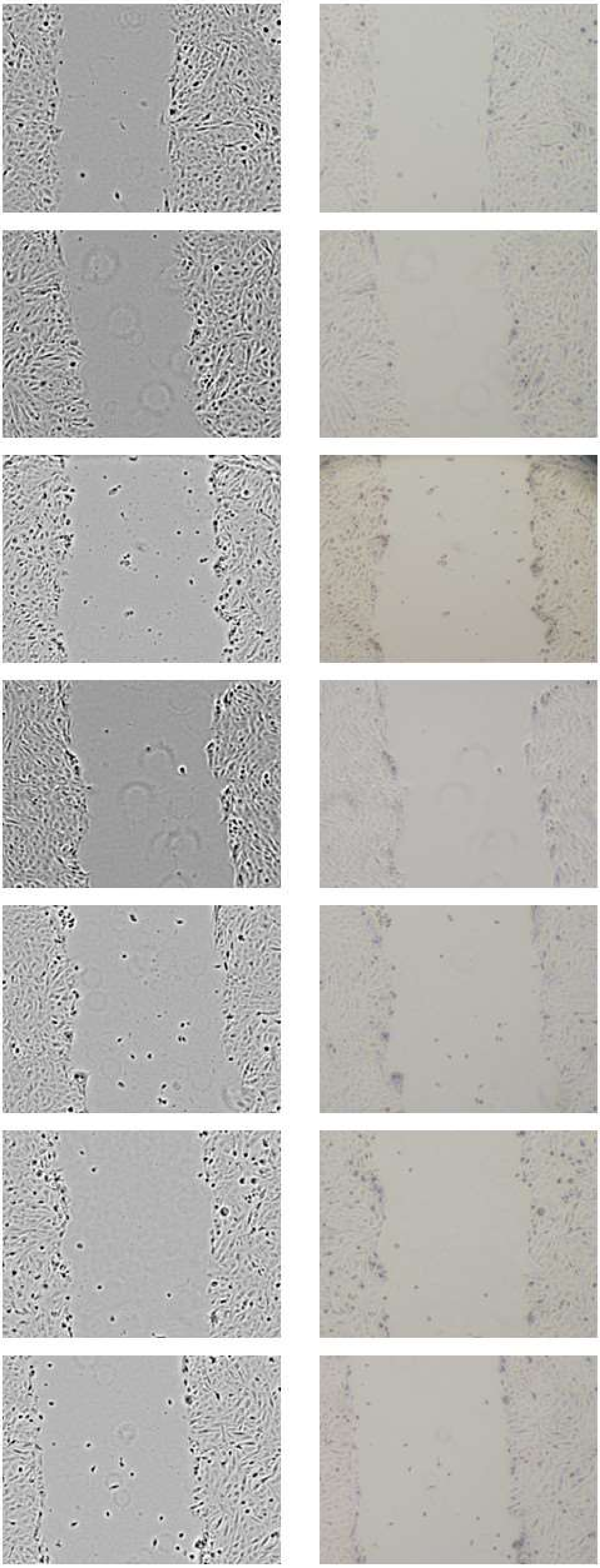

8H

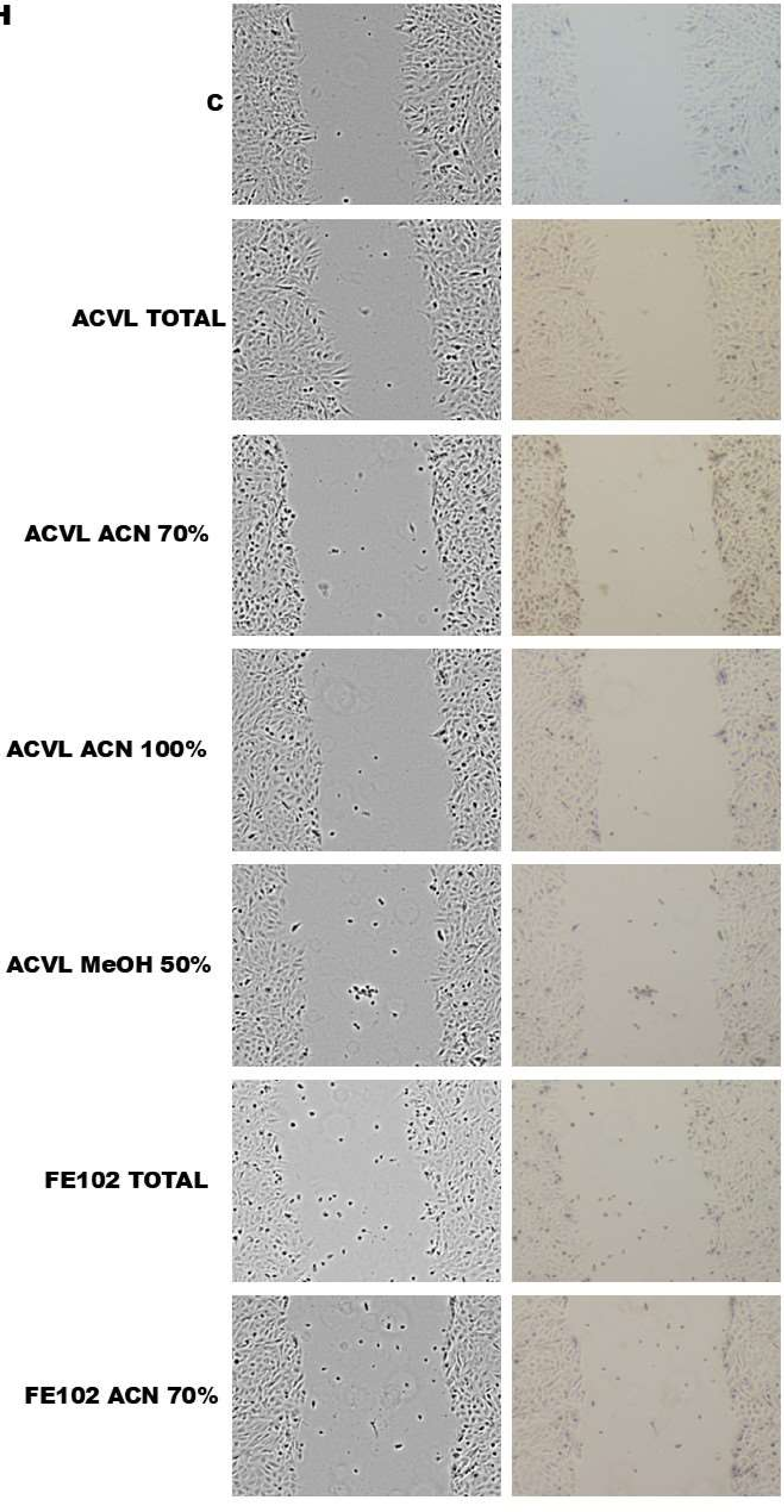
C
ACVL TOTAL
ACVL ACN 70%
ACVL ACN 100%
ACVL MeOH 50%
FE102 TOTAL
FE102 ACN 70%

15h

C

ACVL TOTAL

ACVL ACN 70%

ACVL ACN 100%

ACVL MeOH 50%

FE102 TOTAL

FE102 ACN 70%

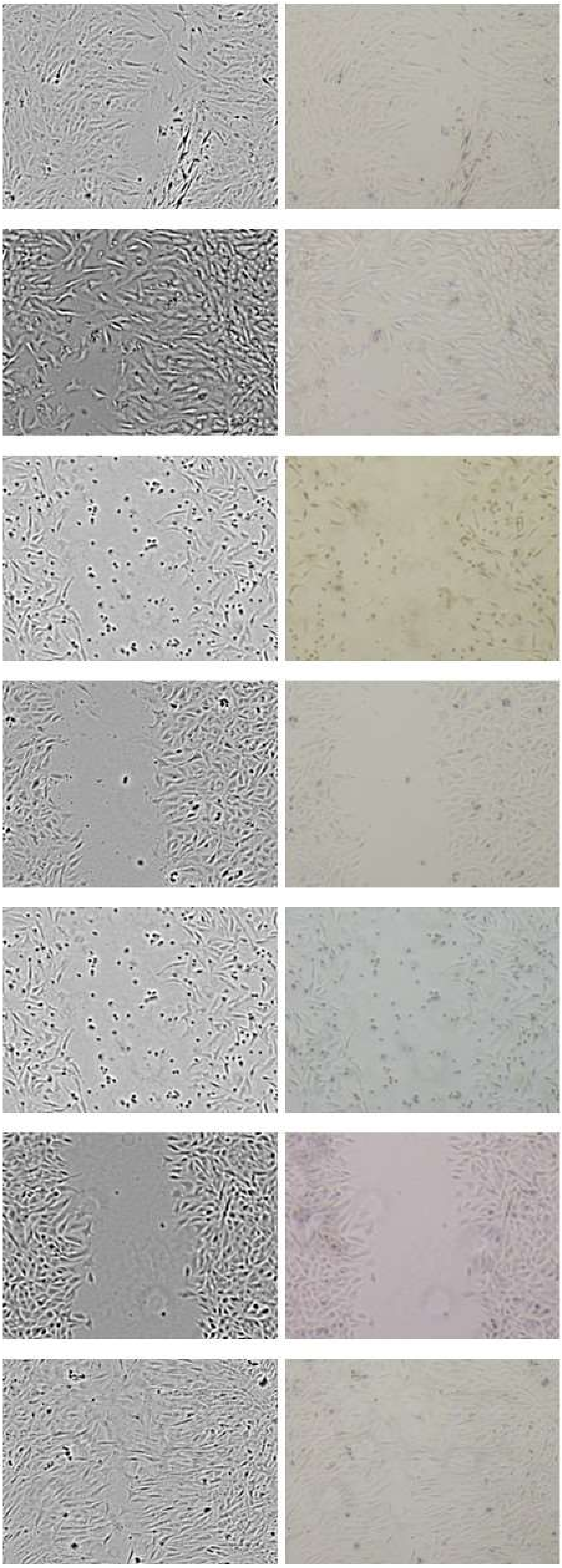

18h

C

ACVL TOTAL

ACVL ACN 70%

ACVL ACN 100%

ACVL MeOH 50%

FE102 TOTAL

FE102 ACN 70%

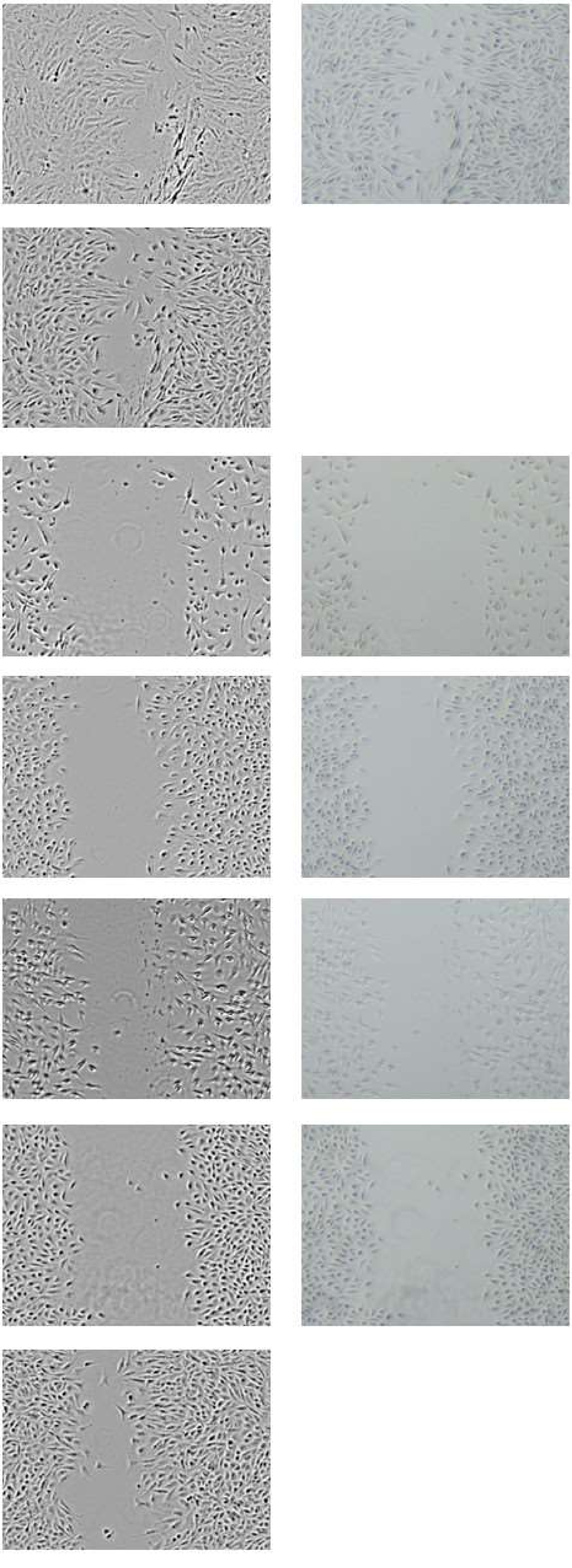

24H

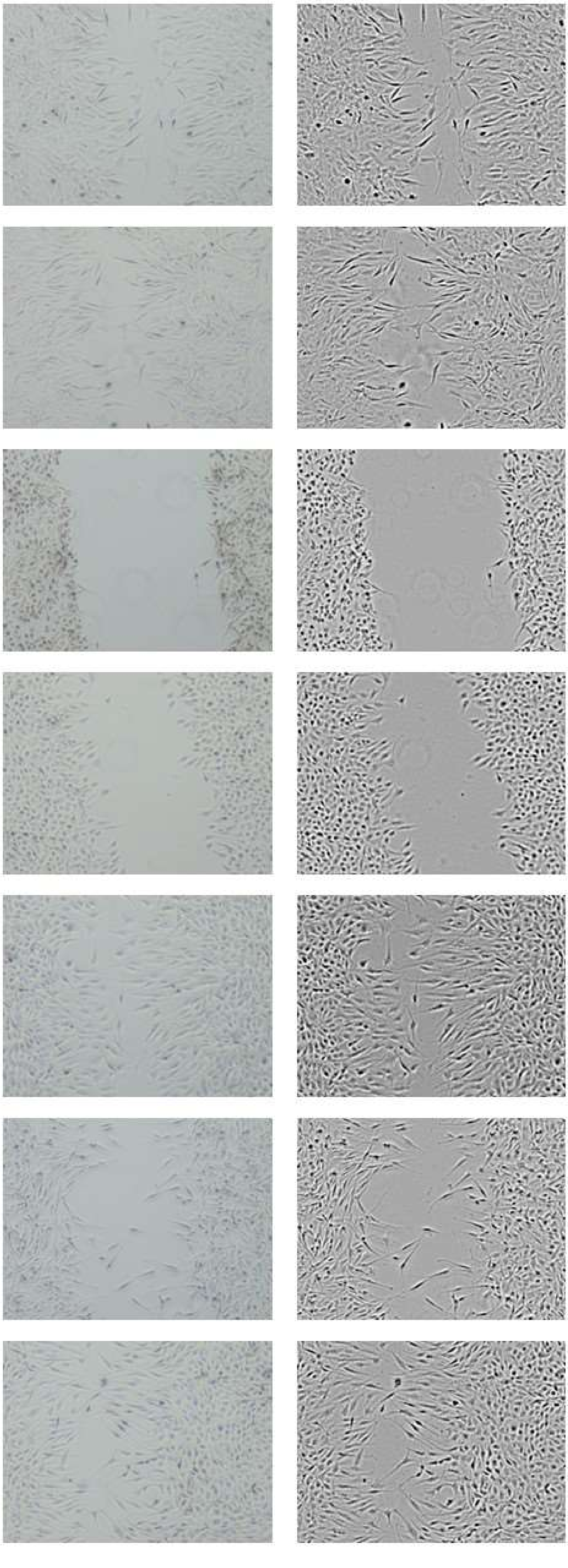

C

ACVL TOTAL

ACVL ACN 70%

ACVL ACN 100%

ACVL MeOH 50%

FE102 TOTAL

FE102 ACN 70%

Original Western Blotting Images

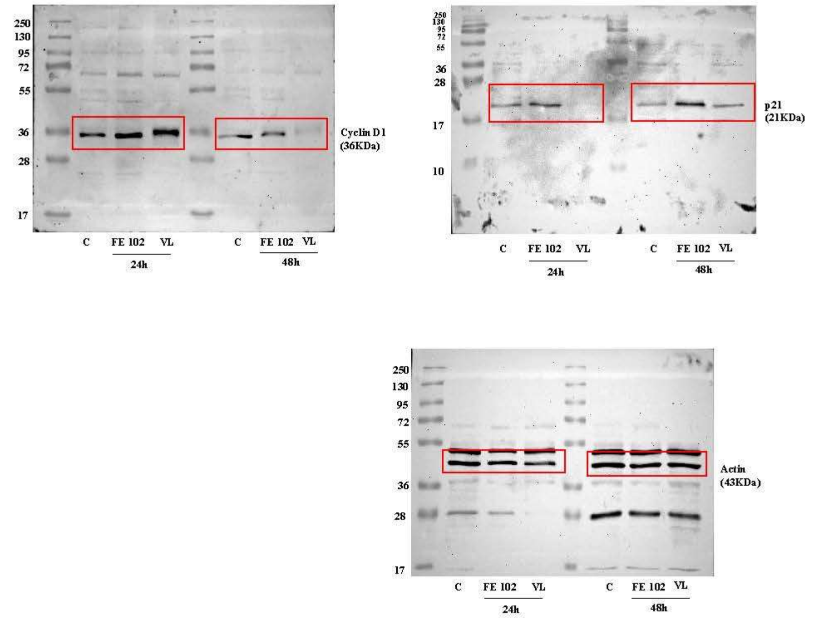

Figure 3

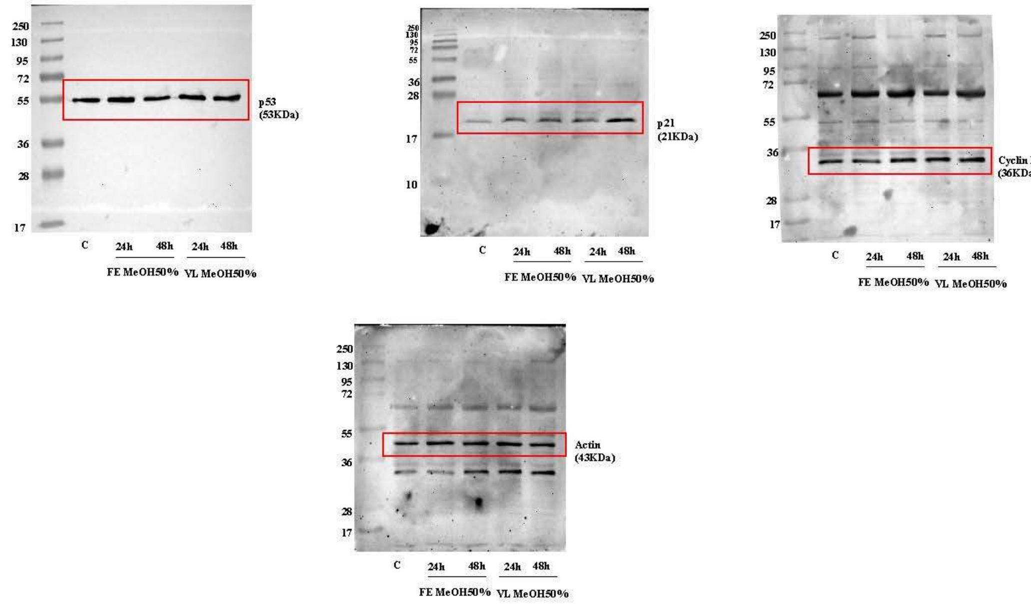

Figure 4

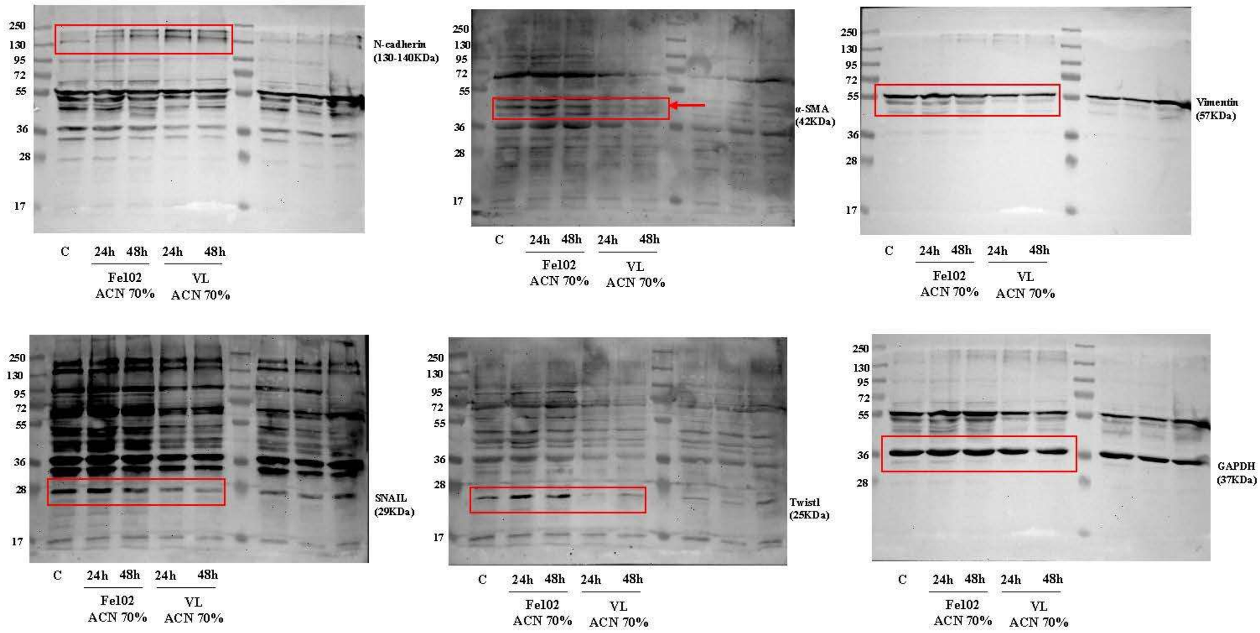

Figure 5

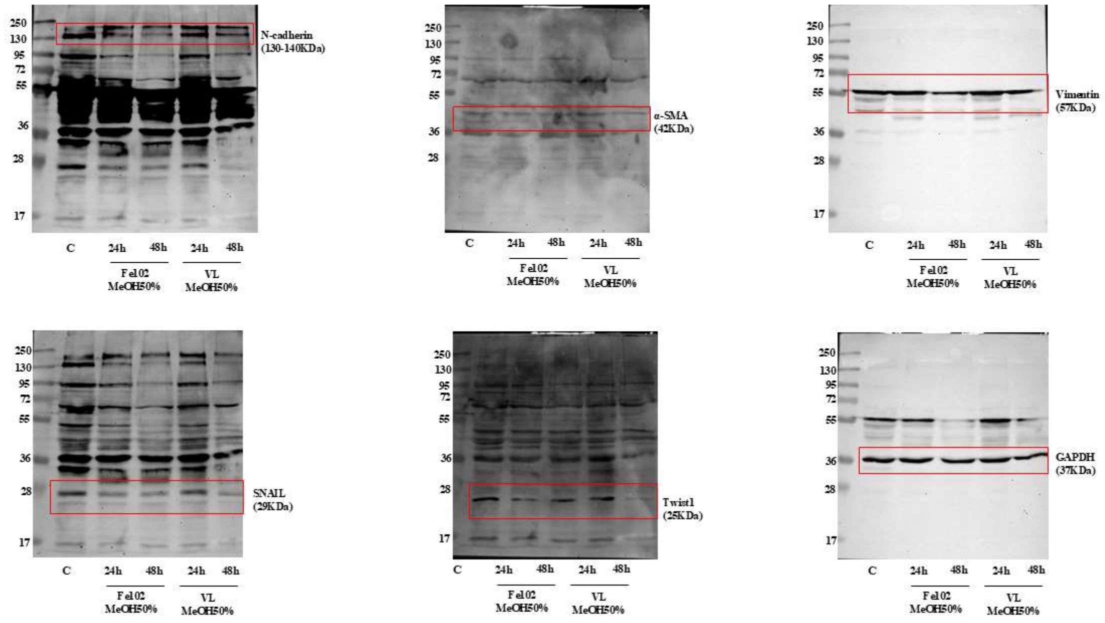

Figure 6

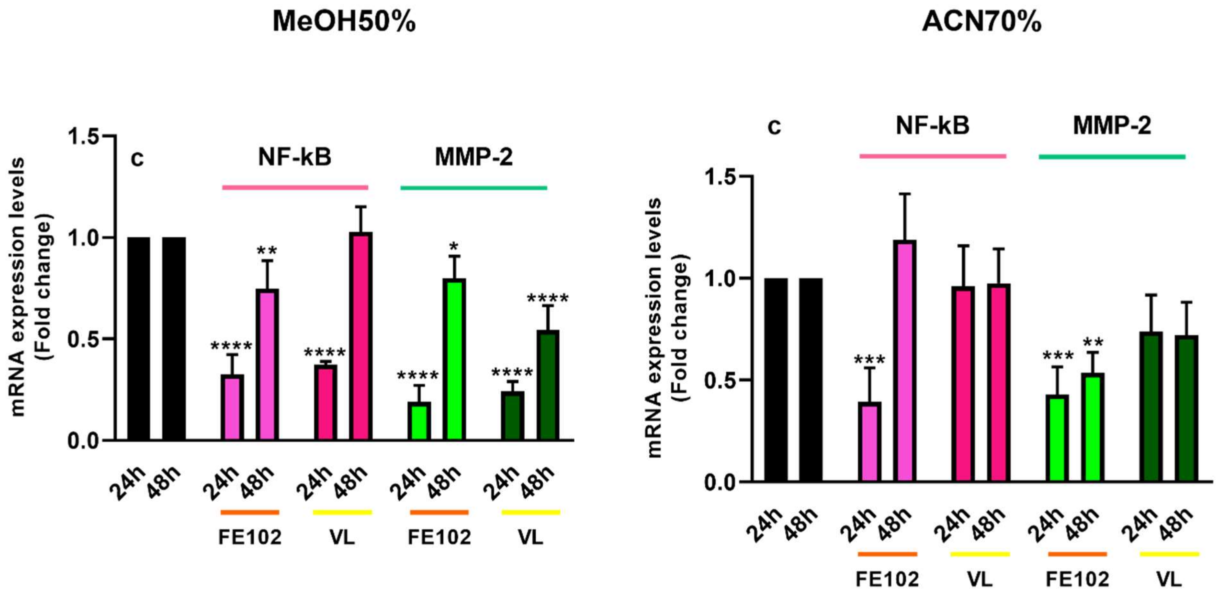

Figure S5. NF-kB and MMP-2 gene expression. qPCR amplification of NF-kB and MMP-2 in cells treated with MeOH50% and ACN70% fractionation of FE102 and VL *A. carterae* strains. Expression was analysed after 24 and 48 hours of treatment. Values are reported as Fold change on not treated cells considered as Control. Refer to main text for methods. Oligos utilized: NF-kB gene amplification: Fw_5'-GAGGTCACTCTAACGTATGCAACAG-3'; Rw_ 5'-TTGCAAGCTGCATAGCCTTCT-3'. MMP-2 gene amplification: Fw: 5'-TGCTGCACCTGGGATTAAGG-3'; Rw: 5'-CACCTTTTGCTCCACGTGC-3'.
Statistical analysis was performed by two-way ANOVA, and the Bonferroni post hoc test was used to compare data (*p<0,05 **p<0.005; ***p<0.0005; ****p<0,0001). Significant differences, for each gene/protein, are reported only versus Control.

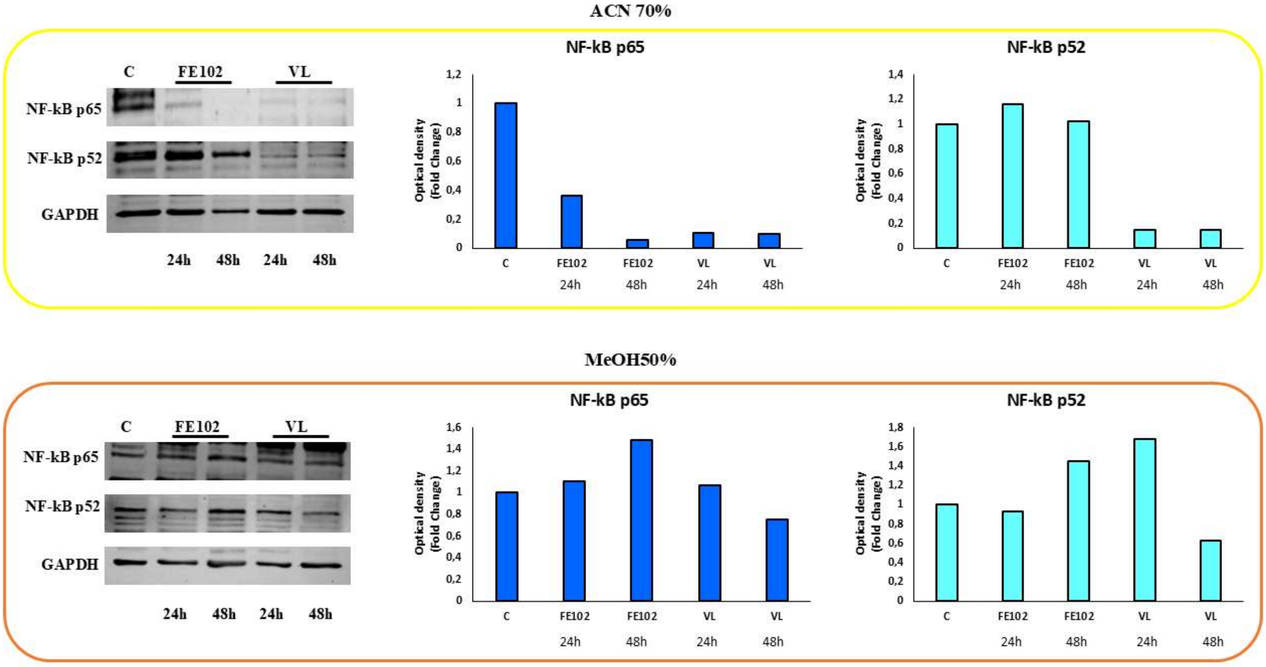

Figure S6. NF-kB protein levels. NF-kB protein levels determined by western blotting in T98G cells treated for 24 and 48 hours with ACN70% and MeOH50% fractionation of the FE102 and VL *A. carterae* strains. Optical densities have been calculated on the corresponding bands derived from the WB hybridization and expressed as fold change ± S.D. relative to Control (not treated cells) after normalization on the GAPDH internal reference protein levels. Refer to the main text for the methods. Antibody utilized are the following: p52_NF-kB: sc-7386; p65_NF-kB: sc-8008 from Santa Cruz Biotechnology, *Inc.* (10410 Finnell Street Dallas, Texas 75220 U.S.A.).

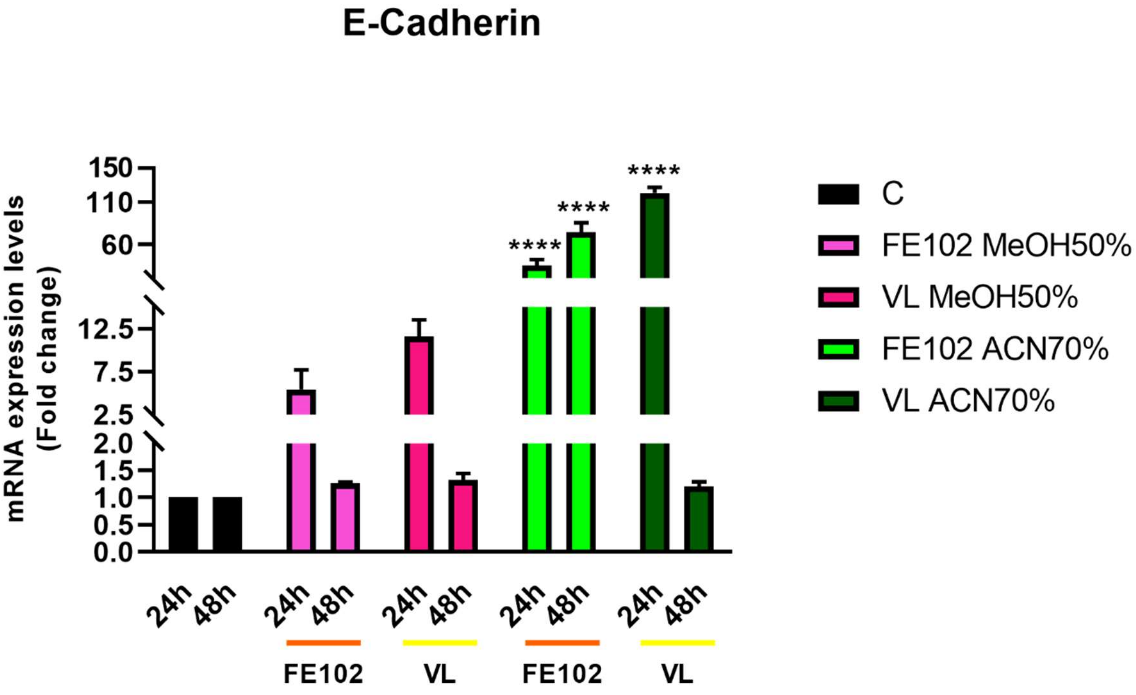

Figure S7. E-Cadherin gene expression. qPCR amplification of E-Cadherin in cells treated with MeOH50% and ACN70% fractionation of FE102 and VL *A. carterae* strains. Expression was analysed after 24 and 48 hours of treatment. Values are reported as Fold change on not treated cells considered as Control. Refer to main text for methods. Oligos utilized for E-Cadherin gene amplification: Fw: 5'-TGCCCAGAAAATGAAAAAGG-3'; Rw: 5'-GTGTATGTGGCAATGCGTTC-3'.

Statistical analysis was performed by two-way ANOVA, and the Bonferroni post hoc test was used to compare data (*p<0,05 **p<0.005; ***p<0.0005; ****p<0,0001). Significant differences, for each gene/protein, are reported only versus Control.
